# A Novel Strategy for Unveiling Spatial Distribution Pattern of Gallotannins in *Paeonia rockii* and *Paeonia ostii* Based on LC–QTRAP–MS

**DOI:** 10.3390/metabo12040326

**Published:** 2022-04-04

**Authors:** Zhangzhen Bai, Rui Yu, Tiantian Zheng, Daoyang Sun, Yang Zhou, Junman Tang, Huili Zhu, Guangning Li, Lixin Niu, Lu Cui, Rui Du, Jing Zhang, Yanlong Zhang

**Affiliations:** 1 National Engineering Technology Research Center for Oil Peony, College of Landscape Architecture and Arts, Northwest A&F University, Yangling 712100, China; zhengtiantian@nwafu.edu.cn (T.Z.); sundaoyang@nwafu.edu.cn (D.S.); zhou_yang@nwafu.edu.cn (Y.Z.); tangjunman@nwafu.edu.cn (J.T.); niulixin@nwafu.edu.cn (L.N.); 2 College of Horticulture, China Agricultural University, Beijing 100193, China; yurui@cau.edu.cn; 3 College of Horticulture, Northwest A&F University, Yangling 712100, China; mega_bisharp@nwafu.edu.cn; 4 SCIEX China, Beijing 100015, China; yououoy@gmail.com; 5 College of Food Science and Engineering, Northwest A&F University, Yangling 712100, China; cuiluctl@nwafu.edu.cn; 6 College of Innovation and experiment, Northwest A&F University, Yangling 712100, China; durui000@nwafu.edu.cn

**Keywords:** gallotannins, tree peony, liquid chromatography-quadrupole ion trap-mass spectrometry (LC–QTRAP–MS), neutral loss, multiple reaction monitoring, spatial distribution pattern

## Abstract

Gallotannins (GTs) are a series of hydrolyzable tannins with multiple health-promoting effects. In this study, an integrated liquid chromatography tandem mass spectrometry (LC–MS/MS) strategy was developed for unveiling the spatial distribution pattern of GTs in the emerging oilseed crops *Paeonia rockii* and *P. ostii*. According to the fragmentation behavior of the representative GT (1,2,3,4,6-penta-*O*-galloyl-β-*D*-glucose, PGG), the diagnostic neutral loss (NL) of 170 Da was chosen for the non-targeted screening of GT precursors. Simultaneously, the tandem mass spectrometry spectrum (MS/MS) information was acquired through an enhanced product ion (EPI) scan. Nine major GTs were identified in tree peony. To quantify the targeted GTs in different tissues of tree peony, we established a multiple reaction monitoring (MRM)–enhanced product ion (EPI)-based pseudo-targeted approach under the information-dependent acquisition (IDA) mode. The quantitative results show that the GT compounds were ubiquitous in tree peony plants with diverse structures. The typical GT PGG was mainly distributed in roots, leaves, and petals. This strategy can also be utilized for metabolite characterization and quantification in other substrates.

## 1. Introduction

Tannins are an important class of phenolic compounds, which can be divided into condensed tannins and hydrolyzable tannins for their hydrolytic properties. Gallotannins (GTs) are a series of hydrolyzable tannins comprising a glucose core surrounded by several galloyl moieties in esterified form. GTs are widespread in dicotyledonous plants, including *Galla chinensis*, *Oenothera paradoxa*, *Hamamelis virginiana*, *Arctostaphylos uva-ursi,* and *Paeonia suffruticosa*, and are widely distributed in fruits, pods, seeds, leaves, barks, and roots [1,2,3,4,5]. Due to their diverse biological properties, such as antioxidant [6], antibacterial [7], anti-inflammatory [8], antiobesity [9], antidiabetic [10], and cardioprotective activities [11], GTs have received much attention as promising bioactive substances in the food, nutraceutical, pharmaceutical, and cosmetic fields.

*P. rockii and P. ostii*, belonging to *Paeonia* Sect. Moutan, are officially authorized species widely cultivated in China for the production of tree peony seed oil (TPSO) [12]. TPSO and tree peony flower are new food resources with various health-promoting effects, which were approved by the National Health Commission of the People’s Republic of China [13,14]. It is reported that GTs, such as 1,2,3,4,6-penta-*O*-galloyl-β-*D*-glucose (PGG), are major bioactive substances in the peony root cortex [15], which is a famous traditional Chinese medicine listed in the Chinese Pharmacopoeia China [16]. However, previous investigations of tree peony GTs have mostly focused on medicinal roots, while comprehensive GT profiles and their spatial distribution pattern in the whole tree peony plant are still unclear. Therefore, unveiling the spatial distribution pattern of GTs in the whole plant of tree peony is crucial for bioactive GTs discovery in the byproduct of oilseed crops.

Alternative esterified sites and the diverse number of galloyl groups lead to the complexity of GT structures. The routine strategy for compound characterization requires a purified monomer acquired through nuclear magnetic resonance (NMR), which is a time-consuming workflow [17]. High-performance liquid chromatography equipped with a tandem mass spectrometer (HPLC–MS/MS) has gradually become an efficient and indispensable tool for profiling metabolites [18]. Non-targeted, full-scan methods, such as time of flight (TOF) and Orbitrap, are commonly used in metabolite analysis [19]. Although these instruments possess the advantage of high resolution, the acquired result has poor linearity, which limits their usage in the accurate quantification of metabolites [20]. There is another method called targeted metabolite analysis, which is performed on a triple quadrupole mass spectrometer (QQQ–MS). It can purposefully select ions in a narrow scan scope in multiple reaction monitoring (MRM) mode, which enhances the linearity for metabolite quantification [21,22]. However, this targeted approach is usually used for known metabolite analysis relying on chemical standards. Therefore, it is still a challenge to identify and quantify unknown metabolites or known metabolites without available standards.

**Figure 1:**
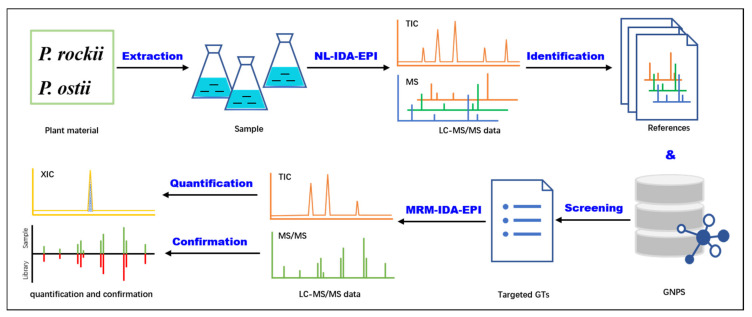
The workflow of the HPLC–QTRAP–MS-based integrated strategy for unveiling the spatial distribution pattern of gallotannins in tree peony. Neutral loss-information-dependent acquisition-enhanced product ion, NL–IDA–EPI; total ion chromatograph, TIC; extracted ion chromatograph, XIC; mass spectrometry spectra, MS; tandem mass spectrometry spectra, MS/MS; multiple reaction monitoring-information-dependent acquisition-enhanced product ion, MRM–IDA–EPI; Global Natural Products Social platform, GNPS.

In this study, we firstly developed a systemic workflow for comprehensively profiling and quantifying GT metabolites in different tissues of *P. rockii* and *P. ostii* with an integrated liquid chromatography-quadrupole ion trap-mass spectrometry (LC–QTRAP–MS) strategy (Figure 1). The major components of this research include (1) fragmentation behavior analysis of GT metabolites, (2) screening and identifying candidate GT precursor ions via neutral loss-information-dependent acquisition-enhanced product ion (NL–IDA–EPI) analysis, (3) optimizing the multiple reaction monitoring (MRM) condition of screened GT compounds using flow injection analysis (FIA), and (4) simultaneously quantifying and confirming targeted GT metabolites through multiple reaction monitoring-information-dependent acquisition-enhanced product ion (MRM–IDA–EPI) analysis. Our study firstly unveiled the spatial distribution pattern of GTs in tree peony plants. This chemical-standard-independent strategy can also be utilized for metabolite identification and quantification in other plant species.

## 2. Results and Discussion

### 2.1. Fragmentation Behaviors of Gallotannins

**Figure 2:**
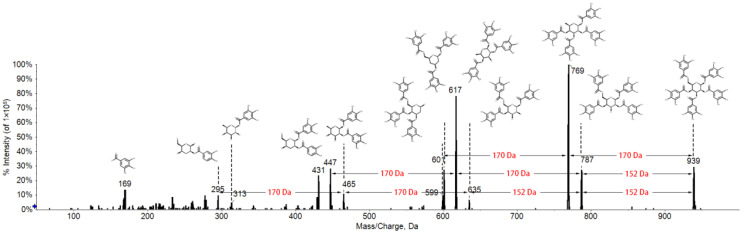
The fragmentation behavior of 1,2,3,4,6-penta-*O*-galloyl-beta-*D*-glucose (PGG).

To investigate the GTs in tree peony for metabolite identification and LC–MS analytical strategy determination, the typical GT 1,2,3,4,6-penta-*O*-galloyl-β-*D*-glucose (PGG) was chosen for fragmentation behavior analysis, which serves as an important drug candidate for treating or preventing various diseases, such as cancer, inflammation, and diabetes [23,24,25]. The collision energy (CE) is a major factor influencing the degree of fragmentation. The MS2 spectrum of PGG was acquired by manually injecting the standard into a QTRAP mass spectrometer with a CE range from 0 to −50 V. When the CE was at −40 V, the MS2 spectrum of PGG became more abundant (Figure 2). The parent ion *m/z* 939 was generated by losing a proton. Fragment ions *m/z* 787, 769, 635, 617, 601, 465, 447, 431, and 169 were the characteristic product ions, which was consistent with previous studies [15,26]. Through an integrative analysis of the molecular structure and MS/MS spectrum on PeakView 1.2 software, we found that the characteristic product ions were produced by the continual loss of neutral ions 152 Da or 170 Da (Figure 2). To further verify whether other gallotannins in tree peony could show similar fragmentation behavior, the LightSight 2.2.1 software was employed. As expected, common losses of 152 Da or 170 Da were frequently observed in all other gallotannins in tree peony (Supplementary File S1), which was in accordance with the published literature [27,28].

### 2.2. Optimization of Chromatographic Conditions for LC–QTRAP–MS Analysis

Metabolites are difficult to separate because of complex compositions and similar physicochemical properties in plants. Chromatographic conditions, including the chromatographic column, column temperature, mobile phase, flow rate, and gradient elution, contribute greatly to the separation efficiency of metabolites. In the optimizing experiment, the QC sample, which contained all of the information about GT metabolites, was used for the improvement of chromatographic separation. Phase A (0.1% formic acid in ultrapure water) and phase B (acetonitrile) were selected as mobile phases for their high separation effects and low baseline in the detection of gallotannins [27]. Compared with the AQ-C18 column (4.6 mm × 150 mm, 5 μm, Shimadzu Corp., Tokyo, Japan), the Kinetex F5 column (3.0 × 100 mm, 2.6 μm) (Phenomenex, Los Angeles, CA, USA) exhibited a better resolution and peak shape. To further improve separation efficiency and shorten the detection time of gallotannins in tree peony, the linear gradient elution procedure was optimized by adjusting the volume ratio of phase A to phase B with consideration of DAD and TIC chromatograms. The final optimized chromatographic conditions of HPLC were performed as described in the Methods Section, and the final optimized effects are shown in Figure S1. The base peak chromatogram (BPC) of the QC sample acquired from MRM–IDA–EPI analysis further proved the chromatographic separation effect of GT compounds (Figure S2).

### 2.3. NL–IDA–EPI for GT Metabolite Identification and Library Construction

Compared with the full scan method, the NL scan can efficiently select targeted precursor ions with the advantages of lower noise and better resolution [29,30]. An enhanced product ion scan (EPI) facilitates the achievement of a high-quality MS/MS spectrum by the enrichment of product ions in the linear ion trap [31]. Due to the fragmentation behavior of GTs characterized by losing 170 Da, we established an untargeted NL–IDA–EPI strategy for the comprehensive profiling of gallotannins in tree peony. To improve efficiency, the mixed sample QC, containing all of the chemical information of tree peony, was used in one run.

**Figure 3:**
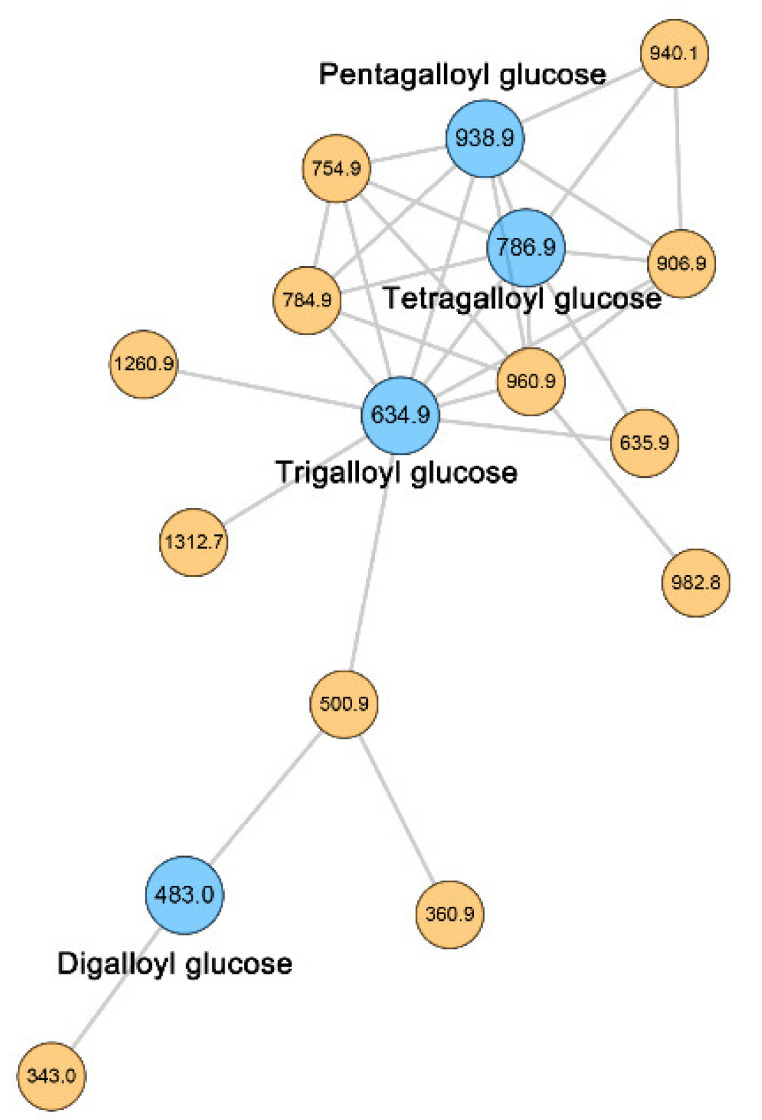
Molecular network of gallotannins in *P. rockii* and *P. ostii* based on NL–IDA–EPI scan.

The identification of tree peony GTs was mainly performed on the GNPS platform using an online workflow. GNPS is an open-access MS/MS library with links to other available online resources, such as MassBank, ReSpect, and METLIN, which facilitate metabolite characterization [32,33,34,35]. The molecular networking of GTs was generated based on the MS/MS spectra similarity. We obtained 77 precursor signals with MS/MS spectra. A total of 36 of them were distributed in the molecular network and classified into six clusters (nodes > 2) (https://gnps.ucsd.edu/ProteoSAFe/staus.jsp?task=b86aa3158d9649b3abda090d4f766b09) on April 19, 2021. A 16-node cluster with four identified GTs—namely, digalloyl glucose (*m/z* 483.1), Trigalloyl glucose (*m/z* 635.1), tetragalloyl glucose (*m/z* 787.1), and pentagalloyl glucose (*m/z* 939.1)—was assigned as the cluster of GTs (Figure 3). To further verify the identification of GT metabolites above, the accurate *m/z* values of parent ions and fragmentation ions were obtained through the full scan of QTOF–MS (Supplementary File S2). By comparing MS/MS spectra with a tolerance of 0.5 Da, all the identified GT metabolites were confirmed. Interestingly, we observed another GT called glucogallin, producing precursor ions of *m/z* 331.0665 and the main product ions of *m/z* 169.0144, 151.0037, and 125.0245, in QTOF data. This compound has been reported in tree peony flower [28]. Thus, five types of GTs were identified on the Global Natural Products Social (GNPS) platform via molecular networking and other general methods.

As most chemical standards of identified GTs are not commercially available, only the retention time (RT) of pentagalloyl glucose was determined. To obtain the RTs of other GTs, the MS data were imported into LightSight 2.2.1 software by comparing MS/MS spectra and the fragmentation pattern of reference GT (PGG). The results show that there were many isomers with different retention times, such as digalloyl glucose and tetragalloyl glucose (Table 1 and Table S1), indicating the high resolution of the established chromatographic separation. Finally, a high-quality GT library, including nine typical GTs, was constructed, which is favorable for the simultaneous identification and quantification of GTs in tree peony via a targeted LC–MS approach (Table 1).

### 2.4. MRM–IDA–EPI for the Simultaneous Identification and Quantification of GTs in P. rockii and P. ostii

Currently, the MRM mode is widely used in targeted metabolomics for its sensitivity and accuracy [36]. Based on the MS2 library of typical GT metabolites, the precursor ion and more than one diagnostic product ions of each targeted compound were screened for MRM analysis (Table S1). To maximize the signals of each ion pair (Q1–Q3), the parameters of DP and CE are usually optimized with chemical standards. However, only one monomer of PGG in the MS2 library was commercially available. To solve this problem, we developed a chemical standard-independent strategy. The parameters of DP and CE can be automatically optimized using identified endogenous metabolites in schedule MRM mode through the flow injection analysis (FIA) method in one run. The optimization results are summarized in Table S1. To avoid false-positive results caused by a limited number of ion pairs, an EPI scan triggered by IDA was also conducted for metabolite confirmation by searching the MS2 library. Therefore, this integrated MRM–IDA–EPI approach has the advantage of simultaneously identifying and quantifying targeted metabolites.

To achieve the true quantification result of PGG in tree peony, a calibration curve (y = 472.09x − 7.30 × 10^5^, R^2^ = 0.9983) was established based on the chemical standard of PGG. The limits of detection and quantification were 0.15 μg/mL and 0.29 μg/mL, respectively. Intraday (RSD) and interday relative standard deviations (RSDs) were 2.19% and 2.28%, respectively. The recovery rate was 96.4% with an RSD of 1.47%. The coefficient of variation (CV) of the RT was less than 0.98%, and the CV of peak areas for all targeted metabolites was below 5.05% (Table S2). The validation results suggest the good linearity, sensitivity, repeatability, stability, accuracy, and precision of this proposed quantification method.

### 2.5. Spatial Distribution Pattern of Gallotannins in P. rockii and P. ostii

**Figure 4:**
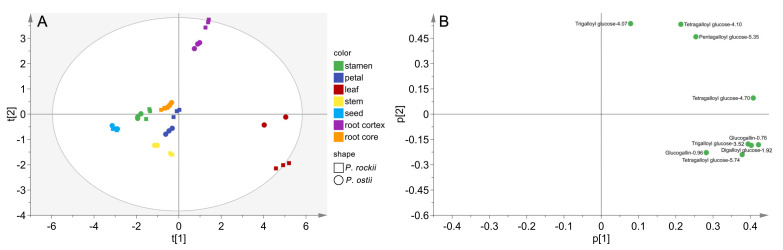
Score plot (**A**) and loading plot (**B**) of principal component analysis of gallotannins in different tissues of *P. rockii* and *P. ostii.*.

**Figure 5:**
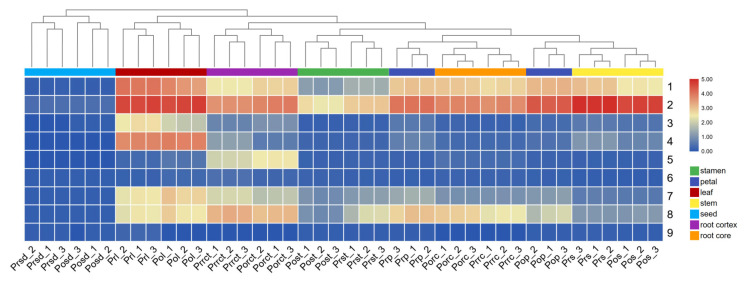
Heatmap of gallotannins in different tissues of *P. rockii* and *P. ostii*.

Based on the integrative strategy of NL–IDA–EPI coupled with MRM–IDA–EPI, all the gallotannins listed in the library were successfully quantified and confirmed. The PCA was performed to overview the relationship of different parts of *P. rockii* and *P. ostii* based on GT contents (Figure 4). The score plot revealed that gallotannins exhibited a similar distribution pattern in two *Paeonia* species (Figure 4A), which was similar to the HCA results (Figure 5). Evidently, the seven plant materials of two species could be divided into three groups. Root cores, stamens, petals, and stems were clustered together, suggesting their similarity in terms of GTs in both composition and content. Root cortexes and leaves were a significant distance away from this group and formed two separate groups. According to the results of the score plot and loading plot, the levels of all targeted gallotannins in root cortexes and leaves were higher than those in other parts of the tree peony (Figure 4).

**Figure 6:**
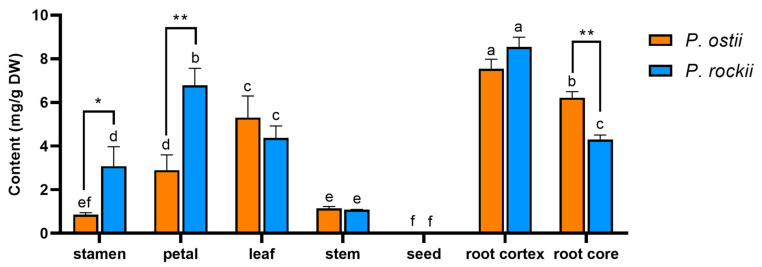
Content of representative gallotannin PGG in different tissues of *P. rockii* and *P. ostii*. * Significant difference at *p* < 0.05 level. ** Significant difference at *p* < 0.01 level. Different letters above the same color column indicate significant differences (*p* < 0.05).

To conduct a quantitative comparison, the contents of each GT in different tissues were summarized, as shown in Figure 5. We observed that GTs were ubiquitous in tree peony with high levels, except in seeds. Glucogallin (or isomer) was the most abundant GT in tree peony. The content of glucogallin at 0.96 min (compound **2**) was much higher than that of glucogallin at 0.76 min (compound **1**) in the corresponding parts of *P. rockii* and *P. ostii*. The highest levels of compound **2** were found in stems, followed by leaves, petals, root cores, root cortexes, stamens, and seeds. Digalloyl glucose was mainly distributed in the leaves of *P. rockii* and *P. ostii*. Two isomers of trigalloyl glucose were detected at 3.52 min (compound **4**) and 4.07 min (compound **5**). These two compounds were mainly accumulated in leaves and root cortexes, respectively. Although three isomers of tetragalloyl glucose were identified in tree peony, tetragalloyl glucose at 4.85 min (compound **7**) was the major existing form when taking content into consideration. Among all the plant materials of *P. rockii* and *P. ostii*, tetragalloyl glucose was mainly distributed in leaves and root cortexes. Compared with other GTs, PGG (compound **8**) was the most frequently reported compound for its diverse biological properties [10,23,24,25]. The contents of PGG in *P. rockii* and *P. ostii* ranged from 0.08 to 8.54 mg/g in dry weight, which was mainly distributed in petals (2.90–6.78 mg/g), leaves (4.60–6.24 mg/g), and roots (4.29–8.54 mg/g) (Figure 6).

**Figure 7:**
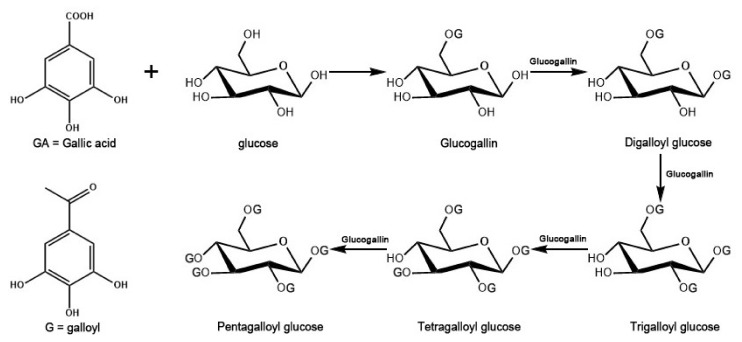
A schematic diagram describing the biosynthetic pathway of gallotannins.

Gallotannins are a type of hydrolyzable tannins, comprising a glucose core surrounded by several esterified galloyl groups. It was revealed that the bioactivities of GTs increased with an increasing number of galloyl groups [26]. Although the biosynthetic pathway of gallotannins has been reported (Figure 7) [37,38], the biosynthetic mechanism of GTs in tree peony is still unclear. The distributed pattern of GTs indicated that the biosynthetic pathways of GTs in different parts of *P. rockii* and *P. ostii* are likely different and complex. The high levels of GT isomers in different parts indicated that the biosynthesis of GTs may be tissue-specific. Therefore, the spatial distribution pattern of GTs could be valuable in unveiling the complicated GT biosynthetic pathway.

## 3. Materials and Methods

### 3.1. Plant Materials

Different plant materials of *P. rockii* and *P. ostii*, including stamens, petals, leaves, stems, root barks, root cores, and seeds, were obtained in the Rare Wild-Flower Resource Nursery of Qinba Mountain, Northwest A&F University, Yangling, China (108.072° E, 34.263° N). Stamens, petals, leaves, stems, root barks, and root cores of tree peony were collected at the early flowering stage, and seeds of tree peony were harvested at maturity. All samples were cleaned and kept at −80 °C before further use.

### 3.2. Chemicals and Reagents

HPLC-grade methanol, acetonitrile, and formic acid were purchased from Merck (Darmstadt, Germany). In addition, 1,2,3,4,6-penta-*O*-galloyl-β-*D*-glucose (PGG) was purchased from Sigma-Aldrich (St. Louis, MO, USA). Ultrapure water was produced using the Milli-Q water purification system (Millipore, Bedford, MA, USA).

### 3.3. Sample Preparation

Plant materials of *P. rockii* and *P. ostii*, including stamens, petals, leaves, stems, root barks, root cores, and seeds, were dried using an LGJ-10D vacuum freeze dryer (Sihuan, Beijing, China) and crushed into fine powder. A total of 0.5 g of the powder was extracted three times with 10 mL of methanol in an ultrasonic bath at 40 kHz for 30 min at room temperature. After centrifugation, the supernatants were mixed and diluted to a fixed volume. Then, the sample solution was filtered through a 0.22 μm membrane before LC–MS analysis. A pool of all sample extracts was used for quality control (QC).

### 3.4. Liquid Chromatography Separation

The liquid chromatographic separation was carried out on the Shimadzu LC-20A system (Tokyo, Japan) equipped with a Kinetex F5 column (3.0 × 100 mm, 2.6 μm) (Phenomenex, Los Angeles, CA, USA). The column oven was maintained at 40 °C. The injection volume of each sample was 2 μL. Solvent A (0.1% formic acid in ultrapure water) and solvent B (acetonitrile) served as mobile phases. An elution gradient mode was used for metabolite separation at a flow rate of 0.4 mL/min with the following procedure: 10% B at 0–1 min; 10%-20% B at 1–3.5 min; 20–30% B at 3.5–8 min; 30–65% B at 8–15 min; 65–98% B at 15–16 min; 98% B at 16–18 min; 98–10% B at 18.1 min; 10% B at 18.1–20 min.

### 3.5. General Parameters of QTRAP–MS

The mass spectrometry analysis was performed on an AB SCIEX QTRAP 5500 platform (Applied Biosystems, Foster, CA, USA) equipped with a turbo electrospray ionization (ESI) source. The Analyst 1.6.3 software (Applied Biosystems, Foster City, CA, USA) was used for system control and data acquisition. All experiments were carried out in negative ion mode. The ion source temperature (T) was set at 600 °C, the ion-spray voltage was set at −4.5 kV. Nitrogen was used in all cases with gas settings of 30 psi for Curtain Gas and 60 psi for ion Source Gas 1 and Source Gas 2. The collision gas was set to high. The entrance potential and collision cell exit potential were set as −10 V and −20 V, respectively. Without specific notes, the MS parameters were the same as described above.

### 3.6. Non-Targeted NL–IDA–EPI Analysis

The neutral loss (NL) scan of the pooled sample was conducted with an NL of 170 Da, and a range from 50 to 1000 Da. The resolution of Q1 and Q3 was set to unit. De-clustering potential (DP) and collision energy (CE) were −60 V and −30 V, respectively. The MS data were obtained by the information-dependent acquisition (IDA) method to select the two most intense peaks. The mass tolerance was set to 0.25 Da. To achieve comprehensive MS2 information, enhanced product ion (EPI) scan mode was performed on a linear ion trap with the same scan range of NL. The resolution of Collision gas was set to high levels. DP was set as −60 V, and CE was set as −40 V with a tolerance of 20.

### 3.7. Targeted MRM–IDA–EPI Analysis

The quantification of GTs for each sample was determined through QQQ in multiple reaction monitoring (MRM) mode. To maximize the signals, the DP and CE of characteristic ion pairs (Q1–Q3) were automatically optimized by FIA in one run. DPs were set as −10, −20, −30, −40, −50, −60, −70, −80, −90, and −100 V. CEs were set as −5, −10, −15, −20, −25, −30, −35, −40, −45, and −50 V. After optimization, the targeted quantification method was established. Additionally, the IDA-triggered EPI scan was carried out for the confirmation of targeted GT compounds, the condition of which was the same as that of non-targeted neutral loss-information-dependent acquisition-enhanced product ion (NL–IDA–EPI) analysis.

### 3.8. ESI–QTOF–MS Analysis

The untargeted ESI–QTOF–MS analysis was performed using LC-30A ultra-performance liquid chromatography (UFLC) (Shimadzu, Kyoto, Japan) coupled with a triple TOF 5600 plus mass spectrometer (AB SCIEX, Foster City, CA, USA). High-resolution MS2 data were acquired via the IDA method with a full scan range from 50 to1500 *m/z*. Other detailed settings were the same as our previous study [39].

### 3.9. Data Processing and Statistical Analysis

The identification of GT metabolites in the pooled sample was performed based on a similar molecular network, which was created using the online workflow on the GNPS website (http://gnps.ucsd.edu) (19/04/2021) [32]. The molecular networking was further visualized using Cytoscape 3.8.2 software. PeakView 1.2 and LightSight 2.2.1 software (AB SCIEX, Foster City, CA, USA) were used for the analysis of fragmentation pattern and GT similarity by comparing MS/MS spectra. MultiQuant 3.0.2 software (AB SCIEX, Foster City, CA, USA) was used for quantification analysis.

The quantification results of GTs are expressed as the mean ± standard deviation (SD). The significance of the data was analyzed by one-way analysis of variance using IBM SPSS 19.0 software (Armonk, NY, USA) at the *p* < 0.05 level. SIMCA 14.1 tools (Umetrics, Umea, Sweden) were used for principal component analysis (PCA). Hierarchical clustering analysis (HCA) and visualization of the heatmap were performed using the TBtools software [40].

## 4. Conclusions

In the present study, we first developed an integrative strategy to simultaneously quantify and confirm the GTs in *P. rockii* and *P. ostii* based on NL–IDA–EPI and MRM–IDA–EPI scans. This novel method is superior to the conventical approach through QTOF–MS or QQQ–MS, which can be applied to the simultaneous identification and quantification of other known or unknown metabolites with identical NL in other plant species. The results unveil the spatial pattern of gallotannins in different parts of tree peony. We observed that glucogallin (or isomer) and PGG were widely present in the whole plant of *P. rockii* and *P. ostii,* apart from the seeds. The typical GT PGG was mainly distributed in petals, leaves, and roots, which has great potential for future utilization in the pharmaceutical, nutraceutical, cosmetic, and food industries. Additionally, the discovery of the spatial distribution pattern of GTs in tree peony provides a helpful clue toward dissecting the complex biosynthetic mechanism of gallotannins.

## Figures and Tables

**Table 1:** Identified gallotannins in *P. rockii* and *P. ostii*.

NO.	RT *	Compound	Formula	[M-H]^−^	Fragment Ions
1	0.76	Glucogallin	C_13_H_16_O_10_	331.1	211, 169, 151, 125
2	0.96	Glucogallin	C_13_H_16_O_10_	331.0	211, 169, 151, 125
3	1.92	Digalloyl glucose	C_27_H_24_O_18_	483.0	313, 271, 211, 169, 125
4	3.52	Trigalloyl glucose	C_27_H_24_O_18_	635.0	465, 313, 169, 125
5	4.07	Trigalloyl glucose	C_27_H_24_O_18_	634.9	483, 465, 313, 169, 125
6	4.20	Tetragalloyl glucose	C_34_H_28_O_22_	787.0	635, 617, 465, 313, 169, 125
7	4.85	Tetragalloyl glucose	C_34_H_28_O_22_	786.7	635, 617, 465, 313, 125
8	5.39	Pentagalloyl glucose	C_41_H_32_O_26_	938.8	787, 769, 617, 601, 599, 169, 125
9	5.74	Tetragalloyl glucose	C_34_H_28_O_22_	786.9	635, 617, 465, 313

* Retention time.

## Data Availability

Data is contained within the article or Supplementary Materials.

## Supplementary Materials

The following supporting information can be downloaded at: https://www.mdpi.com/article/10.3390/metabo12040326/s1, Figure S1: DAD and TIC chromatograms of QC obtained with optimized chromatographic conditions, Figure S2: BPC chromatograms of QC sample acquired from MRM–IDA–EPI analysis, Table S1: Optimized MRM parameters of targeted gallotannins in tree peony, Table S2. The coefficient of variation (CV) of targeted gallotannins, Supplementary File S1: Fragmentation behaviors of gallotannins analyzed by LightSight software, Supplementary File S2: MS/MS information of target gallotannins obtained through QTOF–MS.

## References

[1] Tian F., Li B., Ji B., Yang J., Zhang G., Chen Y., Luo Y. (2009). Antioxidant and antimicrobial activities of consecutive extracts from *Galla chinensis*: The polarity affects the bioactivities. Food Chem..

[2] Kiss A.K., Naruszewicz M. (2012). Polyphenolic compounds characterization and reactive nitrogen species scavenging capacity of *Oenothera paradoxa* defatted seed extracts. Food Chem..

[3] Duckstein S.M., Stintzing F.C. (2011). Investigation on the phenolic constituents in *Hamamelis virginiana* leaves by HPLC-DAD and LC-MS/MS. Anal. Bioanal. Chem..

[4] Panusa A., Petrucci R., Marrosu G., Multari G., Gallo F.R. (2015). UHPLC-PDA-ESI-TOF/MS metabolic profiling of *Arctostaphylos pungens* and *Arctostaphylos uva-ursi*. A comparative study of phenolic compounds from leaf methanolic extracts. Phytochemistry.

[5] Kiss A.K., Piwowarski J.P. (2018). Ellagitannins, gallotannins and their metabolites- the contribution to the anti-inflammatory effect of food products and medicinal plants. Curr. Med. Chem..

[6] Chambi F., Chirinos R., Pedreschi R., Betalleluz-Pallardel I., Debaste F., Campos D. (2013). Antioxidant potential of hydrolyzed polyphenolic extracts from tara (*Caesalpinia spinosa*) pods. Ind. Crop. Prod..

[7] Aguilar-Galvez A., Noratto G., Chambi F., Debaste F., Campos D. (2014). Potential of tara (*Caesalpinia spinosa*) gallotannins and hydrolysates as natural antibacterial compounds. Food Chem..

[8] Erdèlyi K., Kiss A., Bakondi E., Bai P., Szabó C., Gergely P., Erdödi F., Virág L. (2005). Gallotannin inhibits the expression of chemokines and inflammatory cytokines in A549 cells. Mol. Pharmacol..

[9] Li L., Ma H., Liu T., Ding Z., Liu W., Gu Q., Mu Y., Xu J., Seeram N.P., Huang X. (2020). Glucitol-core containing gallotannins-enriched red maple (*Acer rubrum*) leaves extract alleviated obesity via modulating short-chain fatty acid production in high-fat diet-fed mice. J. Funct. Foods.

[10] Li Y., Kim J., Li J., Liu F., Liu X., Himmeldirk K., Ren Y., Wagner T.E., Chen X. (2005). Natural anti-diabetic compound 1,2,3,4,6-penta-O-galloyl-d-glucopyranose binds to insulin receptor and activates insulin-mediated glucose transport signaling pathway. Biochem. Biophys. Res. Commun..

[11] Lee H., Lee J.Y., Suh M.H., Sim S.-S., Lee M.-W., Kim C.J. (2010). Hydrolysable tannins depress cardiac papillary muscle contraction and propranolol-induced negative inotropism. Fitoterapia.

[12] Zhang L., Liu P., Gao J., Wang X., An J., Xu S., Deng R. (2019). Profiling and simultaneous quantitative determination of oligostilbenes in *Paeonia ostii* seed shell from different geographical areas in China and their comparative evaluation. Phytochem. Anal..

[13] Xiang J.L., Yang C.B., Beta T., Liu S.X., Yang R.Q. (2019). Phenolic profile and antioxidant activity of the edible tree peony flower and underlying mechanisms of preventive effect on H_2_O_2_-induced oxidative damage in Caco-2 cells. Foods.

[14] Zhang Y., Liu P., Gao J.-Y., Wang X.-S., Yan M., Xue N.-C., Qu C.-X., Deng R.-X. (2018). *Paeonia veitchii* seeds as a promising high potential by-product: Proximate composition, phytochemical components, bioactivity evaluation and potential applications. Ind. Crop. Prod..

[15] Li S.-S., Wu Q., Yin D.-D., Feng C., Liu Z.-A., Wang L.-S. (2018). Phytochemical variation among the traditional Chinese medicine Mu Dan Pi from *Paeonia suffruticosa* (tree peony). Phytochemistry.

[16] Chinese Pharmacopoeia Commission (2015). The Pharmacopoeia of the People’s Republic of China.

[17] Seger C., Sturm S., Stuppner H. (2013). Mass spectrometry and NMR spectroscopy: Modern high-end detectors for high resolution separation techniques—State of the art in natural product HPLC-MS, HPLC-NMR, and CE-MS hyphenations. Nat. Prod. Rep..

[18] Jiang Y., David B., Tu P., Barbin Y. (2010). Recent analytical approaches in quality control of traditional Chinese medicines—A review. Anal. Chim. Acta.

[19] Dunn W.B., Broadhurst D., Atherton H.J., Goodacre R., Griffin J.L. (2011). Systems level studies of mammalian metabolomes: The roles of mass spectrometry and nuclear magnetic resonance spectroscopy. Chem. Soc. Rev..

[20] Hendriks M.M., Eeuwijk F.A., Jellema R.H., Westerhuis J.A., Reijmers T.H., Hoefsloot H.C., Smilde A.K. (2011). Data-processing strategies for metabolomics studies. TrAC Trends Anal. Chem..

[21] Yuan M., Breitkopf S.B., Yang X., Asara J.M. (2012). A positive/negative ion–switching, targeted mass spectrometry—Based metabolomics platform for bodily fluids, cells, and fresh and fixed tissue. Nat. Protoc..

[22] Chen S., Kong H., Lu X., Li Y., Yin P., Zeng Z., Xu G. (2013). Pseudotargeted metabolomics method and its application in serum biomarker discovery for hepatocellular carcinoma based on ultra high-performance liquid chromatography/triple quadrupole mass spectrometry. Anal. Chem..

[23] Yang J., Wang F., Chen X., Qiu S., Cui L., Hu L. (2019). β-Pentagalloyl-glucose sabotages pancreatic cancer cells and ameliorates cachexia in tumor-bearing mice. Am. J. Chin. Med..

[24] Zhang J., Li L., Kim S.-H., Hagerman A.E., Lü J. (2009). Anti-cancer, anti-diabetic and other pharmacologic and biological activities of penta-galloyl-glucose. Pharm. Res..

[25] Peng J., Li K., Zhu W., Nie R., Wang R., Li C. (2019). Penta-O-galloyl-β-d-glucose, a hydrolysable tannin from *Radix Paeoniae* Alba, inhibits adipogenesis and TNF-α-mediated inflammation in 3T3-L1 cells. Chem. Interact..

[26] Tian F., Li B., Ji B., Zhang G., Luo Y. (2009). Identification and structure—Activity relationship of gallotannins separated from *Galla chinensis*. LWT—Food Sci. Technol..

[27] Dienaitė L., Pukalskienė M., Pukalskas A., Pereira C.V., Matias A.A., Venskutonis P.R. (2019). Isolation of strong antioxidants from *Paeonia Officinalis* roots and leaves and evaluation of their bioactivities. Antioxidants.

[28] He J.Y., Dong Y.Q., Liu X.Y., Wan Y.L., Gu T.W., Zhou X.F., Liu M.H. (2019). Comparison of chemical compositions, antioxidant, and anti-photoaging activities of *Paeonia suffruticosa* flowers at different flowering stages. Antioxidants.

[29] Huang Y.-Q., Wang Q.-Y., Liu J.-Q., Hao Y.-H., Yuan B.-F., Feng Y.-Q. (2014). Isotope labelling—Paired homologous double neutral loss scan-mass spectrometry for profiling of metabolites with a carboxyl group. Analyst.

[30] Steen R.J.C.A., Bobeldijk I., Brinkman U.A.T. (2001). Screening for transformation products of pesticides using tandem mass spectrometric scan modes. J. Chromatogr. A.

[31] Hager J.W., Yves L.B.J. (2003). Product ion scanning using a Q-q-Qlinear ion trap (Q TRAPTM) mass spectrometer. Rapid Commun. Mass Spectrom..

[32] Wang M., Carver J.J., Phelan V.V., Sanchez L.M., Garg N., Peng Y., Nguyen D.D., Watrous J., Kapono C.A., Luzzatto-Knaan T. (2016). Sharing and community curation of mass spectrometry data with Global Natural Products Social Molecular Networking. Nat. Biotechnol..

[33] Horai H., Arita M., Kanaya S., Nihei Y., Ikeda T., Suwa K., Ojima Y., Tanaka K., Tanaka S., Aoshima K. (2010). MassBank: A public repository for sharing mass spectral data for life sciences. Biol. Mass Spectrom..

[34] Sawada Y., Nakabayashi R., Yamada Y., Suzuki M., Sato M., Sakata A., Akiyama K., Sakurai T., Matsuda F., Aoki T. (2012). RIKEN tandem mass spectral database (ReSpect) for phytochemicals: A plant-specific MS/MS-based data resource and database. Phytochemistry.

[35] Domingo-Almenara X., Montenegro-Burke J.R., Ivanisevic J., Thomas A., Sidibé J., Teav T., Guijas C., Aisporna A.E., Rinehart D., Hoang L. (2018). XCMS-MRM and METLIN-MRM: A cloud library and public resource for targeted analysis of small molecules. Nat. Methods.

[36] Chen W., Gong L., Guo Z., Wang W., Zhang H., Liu X., Yu S., Xiong L., Luo J. (2013). A novel integrated method for large-scale detection, identification, and quantification of widely targeted metabolites: Application in the study of rice metabolomics. Mol. Plant.

[37] Grundhöfer P., Niemetz R., Schilling G., Gross G.G. (2001). Biosynthesis and subcellular distribution of hydrolyzable tannins. Phytochemistry.

[38] Denzel K., Schilling G., Gross G.G. (1988). Biosynthesis of gallotannins. Enzymatic conversion of 1,6-digalloylglucose to 1,2,6-trigalloylglucose. Planta.

[39] Bai Z.-Z., Tang J.-M., Ni J., Zheng T.-T., Zhou Y., Sun D.-Y., Li G.-N., Liu P., Niu L.-X., Zhang Y.-L. (2021). Comprehensive metabolite profile of multi-bioactive extract from tree peony (*Paeonia ostii* and *Paeonia rockii*) fruits based on MS/MS molecular networking. Food Res. Int..

[40] Chen C., Chen H., Zhang Y., Thomas H.R., Frank M.H., He Y., Xia R. (2020). TBtools: An integrative toolkit developed for interactive analyses of big biological data. Mol. Plant.

