# Supplementary material for: A Novel Strategy for Unveiling Spatial Distribution Pattern of Gallotannins in Paeonia rockii and Paeonia ostii Based on LC–QTRAP–MS
Source: Metabolites. 2022 Apr 4;12(4):326. doi: 10.3390/metabo12040326 (PMC9030617; doi:10.3390/metabo12040326)
Supplement: Supplementary file 1 [file metabolites-12-00326-s001.zip › Supplementary File S1.pdf]

Report

Generated by LightSight® Software

COMPOUND INFORMATION

Name:	PGG
Formula:	$C_{41}H_{32}O_{26}$
Isotope Pattern Looked For:	Off
Molecular Weight:	940.1 Da
m/z used:	939.1

PROCESSING PARAMETERS

Retention Time Window:	0.01 to 20.01 minutes
Control Trace Offset:	N/A
Parameters Used:	Default Parameters
Sample/Control Peak Area Ratio:	N/A
Peak Separation:	1
Minimum Chromatographic Peak Width:	4.0 seconds
Analog Relative Intensity Threshold:	2%
Analog Signal to Noise Ratio:	5
Analog Signal to Noise Points to Smooth:	3
Analog Retention Time Offset:	0.00
Analog Chromatographic Alignment:	0.00
Selected Wavelength:	190 to 700nm

Search for Unexpected: On

Survey Scan	Exclusion Tolerance	Rolling Background Subtraction
MRM	N/A	N/A
Precursor Ion	0.35	10
Neutral Loss	0.35	10
Q1	0.20	10
Q3	0.20	10
Full Scan EMS	0.20	10

MS Parameters

Survey Scan	S/N Ratio	S/N pt. Smooth	Chrom. Intensity (cps)	Spectral Intensity (cps)
MRM	6.0	3	5000.0	N/A
Precursor Ion	6.0	3	25000.0	25000.0
Neutral Loss	6.0	3	25000.0	25000.0
Q1	6.0	3	100000.0	250000.0
Q3	6.0	3	100000.0	250000.0
Full Scan EMS	6.0	3	100000.0	250000.0

BIOTRANSFORMATION SET

Set used: DANTIEGAN

Biotransformation	Mass Shift	Formula
-Tergalloyl	-608.0	C28H16O16
-Trigalloyl	-456.0	C21H12O12
-Digallic acid	-304.0	C14H808
-Galloyl	-152.0	C7H4O4
Decarboxylation	-44.0	CO2
Loss of H2O	-18.0	H2O
Demethylation	-14.0	CH2
Dehydrogenation	-2.0	H2
Parent	0.0	
Hydrogenation	+2.0	H2
Methylation	+14.0	CH2
Oxidation	+16.0	O
Di-Methylation	+28.0	C2H4
Di-Oxidation	+32.0	O2
Acetylation	+42.0	C2H2O
B	+104.0	C7H4O
HB	+120.0	C7H4O2
Ribose	+132.0	C5H8O4
MG	+134.0	C8H6O2
Va	+150.0	C8H6O3
Galloyl	+152.0	C7H4O4
Glucose	+162.0	C6H10O5
Mannitol	+164.0	C6H12O5

POTENTIAL METABOLITES

Peak ID	Biotransformation	Mass Shift	m/z expected/found	R.T. (min)	Peak Area	% Area	Peak Height	R.T. (min.) Analog	Peak Area Analog	% Area Analog
M5	-digalloyl	-304.0	635.1 / 634.9	4.07	3.38e7	4.6	3.73e6			
M3	-digalloyl	-304.0	635.1 / 635.0	3.52	2.67e8	36.2	3.58e7	3.58	4.67e1	5.6
M6	-galloyl	-152.0	787.1 / 787.0	4.20	2.27e6	0.3	4.40e5			
M11	-galloyl	-152.0	787.1 / 787.1	6.24	1.59e6	0.2	2.79e5			
M10	-galloyl	-152.0	787.1 / 786.9	5.74	3.08e6	0.4	3.56e5	5.74	2.31e2	27.5
M7	-galloyl	-152.0	787.1 / 786.7	4.85	1.58e8	21.3	1.29e7	4.83	2.23e1	2.7
	Parent	0.0	939.1 / 938.8	5.39	1.41e8	19.1	9.53e6	5.32	6.75e1	8.1
M2	trigalloyl	-456.0	483.1 / 483.2	2.22	3.33e6	0.5	3.60e5	2.08	1.15e2	13.8
M1	trigalloyl	-456.0	483.1 / 483.0	1.92	1.71e7	2.3	2.40e6			

PARENT_Pentagalloyl glucose

Parent (0) in experiment #1: -NL(169.90)
Expected m/z: 939.1
Found m/z: 938.8

	Retention Time	Peak Area
MS	5.39	1.41e8
Analog	5.32	6.75e1

CHROMATOGRAM (XIC)

XIC of 939.1 (Parent/0.0)

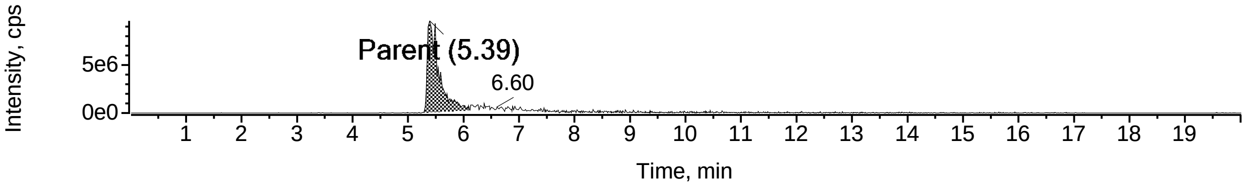

ANALOG CHROMATOGRAM (XWC)

Analog Data

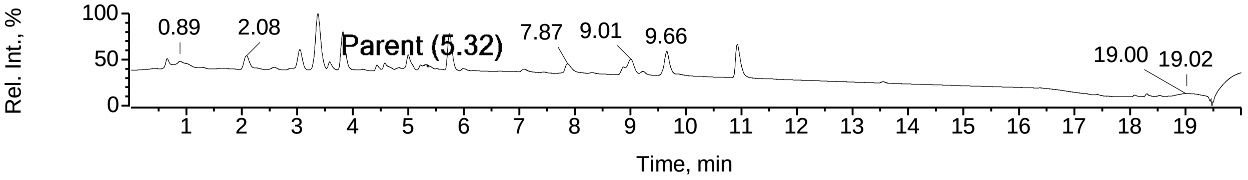

MS SPECTRUM

-MS at 5.39 min

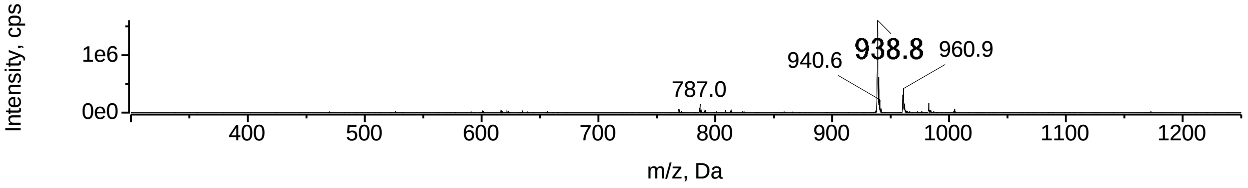

MS/MS COMPARISON WITH PARENT

(Parent Retention Time: 5.38 minutes)

-MSMS of 939.1 (Parent)

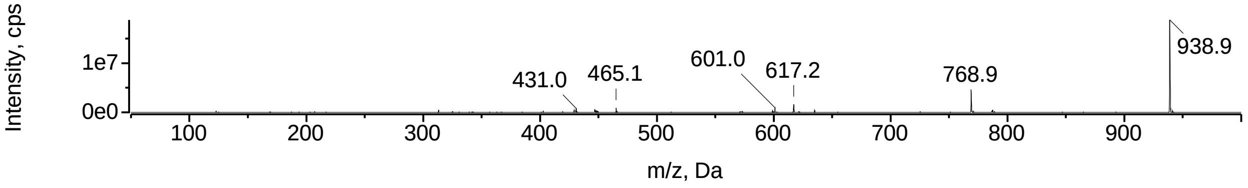

-MSMS of 939.1 (Parent)

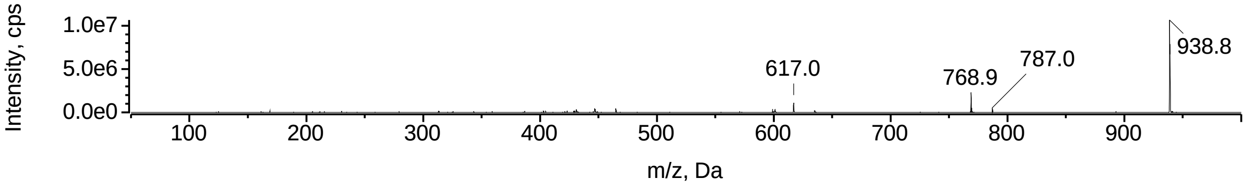

Common product ions (Min. Relative Intensity Shown: 5 %)

617.2, 768.8, 938.8

Common losses (Min. Relative Intensity Shown: 5 %)

Loss	Metabolite m/z	Parent m/z
-321.9	617.2	617.2
-170.3	768.8	768.8
-0.2	938.9	938.8

M5_Trigalloyl glucose

-digalloyl (-304) in experiment #1: -NL(169.90)

Expected m/z: 635.1

Found m/z: 634.9

	Retention Time	Peak Area
MS	4.07	3.38e7

CHROMATOGRAM (XIC)

XIC of 635.1 (-digalloyl/-304.0)

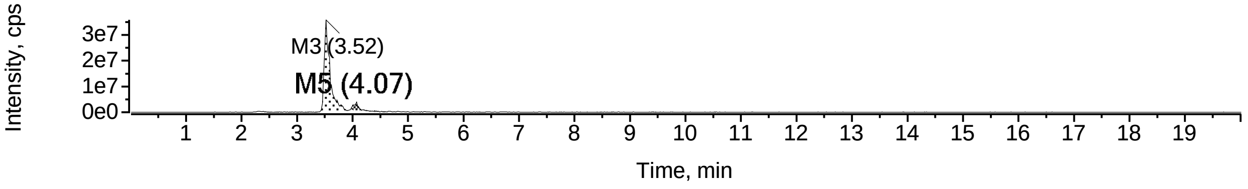

ANALOG CHROMATOGRAM (XWC)

Analog Data

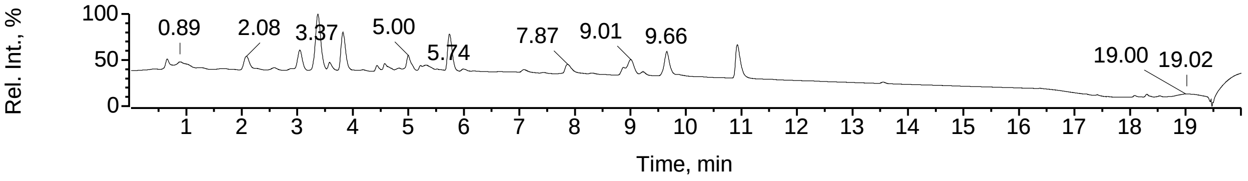

MS SPECTRUM

-MS at 4.07 min

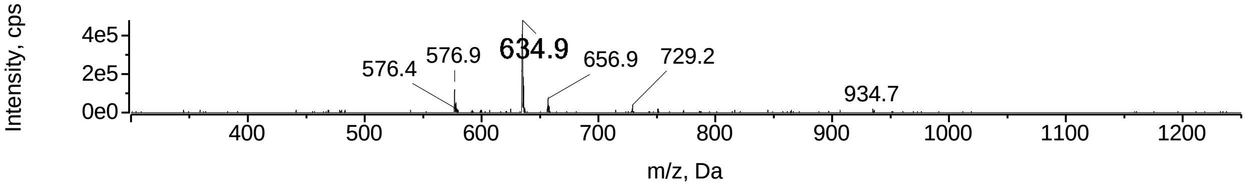

MS/MS COMPARISON WITH PARENT

(Parent Retention Time: 5.38 minutes)

-MSMS of 635.1 (-digalloyl)

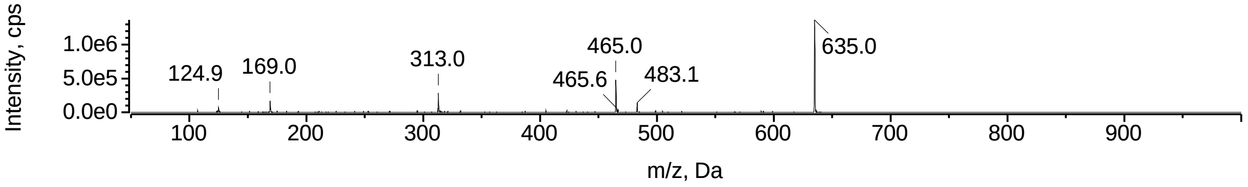

-MSMS of 939.1 (Parent)

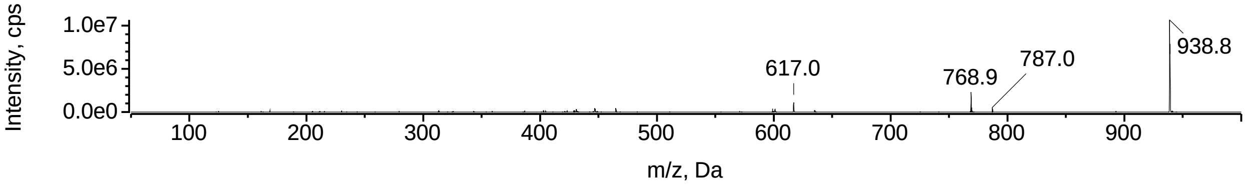

Common product ions (Min. Relative Intensity Shown: 5 %)

168.8, 313.2, 464.8, 634.8

Common losses (Min. Relative Intensity Shown: 5 %)

Loss	Metabolite m/z	Parent m/z
-322.0	313.1	617.2
-170.2	464.9	768.8
-152.0	483.1	787.0
-0.2	634.9	938.8

M3_Trigalloyl glucose

-digalloyl (-304) in experiment #1: -NL(169.90)
Expected m/z: 635.1
Found m/z: 635.0

	Retention Time	Peak Area
MS	3.52	2.67e8
Analog	3.58	4.67e1

CHROMATOGRAM (XIC)

XIC of 635.1 (-digalloyl/-304.0)

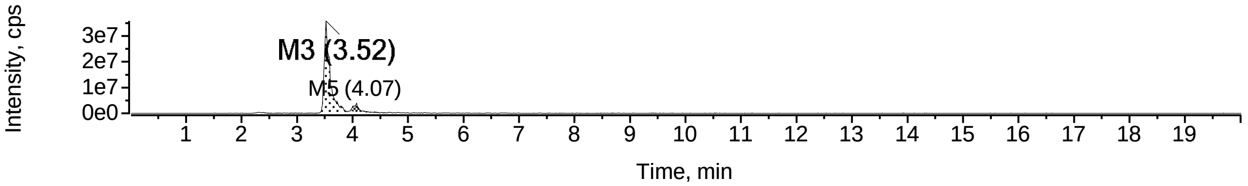

ANALOG CHROMATOGRAM (XWC)

Analog Data

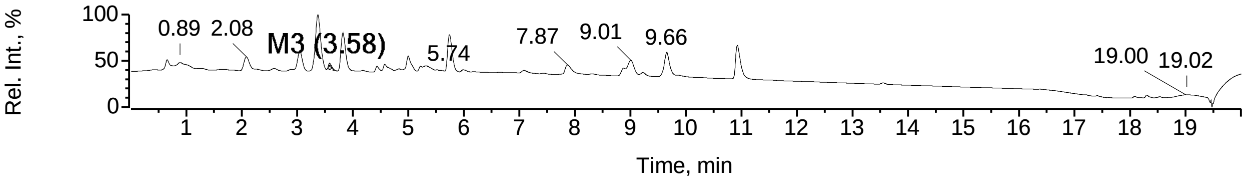

MS SPECTRUM

-MS at 3.52 min

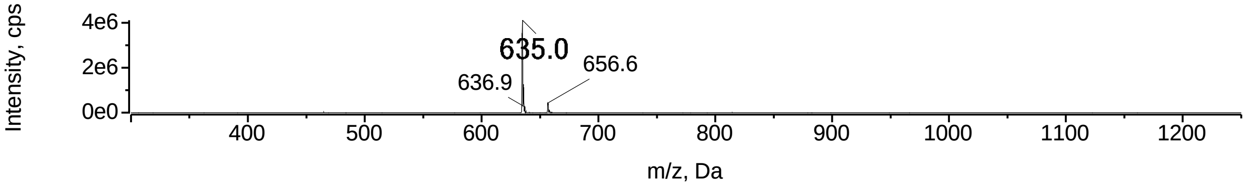

MS/MS COMPARISON WITH PARENT

(Parent Retention Time: 5.38 minutes)

-MSMS of 635.1 (-digalloyl)

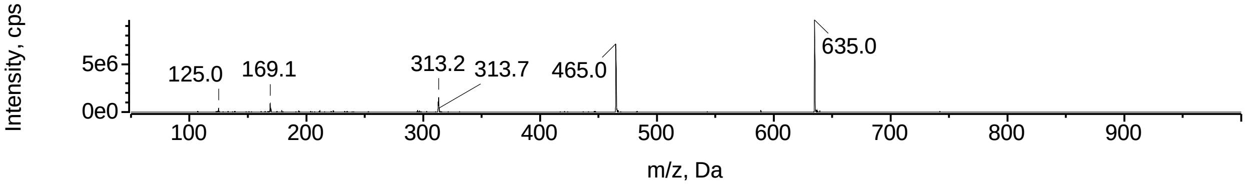

-MSMS of 939.1 (Parent)

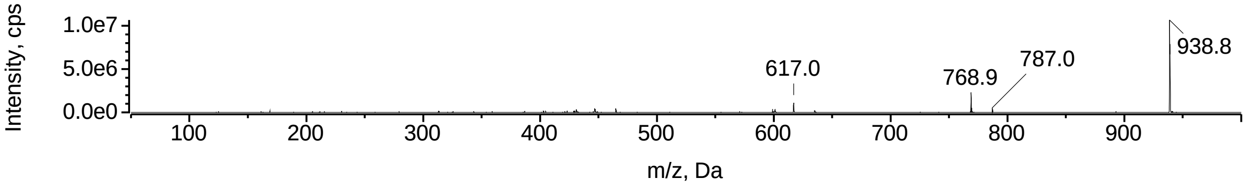

Common product ions (Min. Relative Intensity Shown: 5 %)

Common losses (Min. Relative Intensity Shown: 5 %)

Loss	Metabolite m/z	Parent m/z
-321.8	313.3	617.2
-170.2	464.9	768.8
-0.2	634.9	938.8

M6_Tetragalloyl glucose

-galloyl (-152) in experiment #1: -NL(169.90)
Expected m/z: 787.1
Found m/z: 787.0

	Retention Time	Peak Area
MS	4.20	2.27e6

CHROMATOGRAM (XIC)

XIC of 787.1 (-galloyl/-152.0)

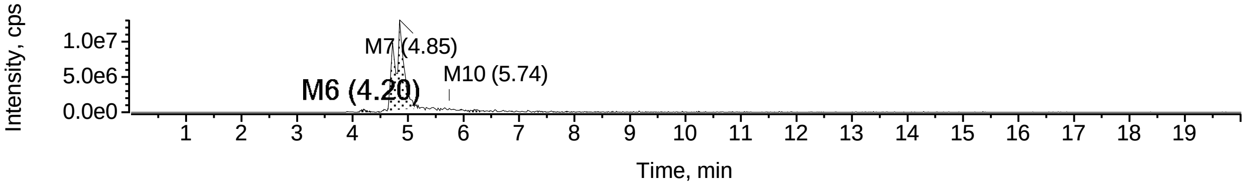

ANALOG CHROMATOGRAM (XWC)

Analog Data

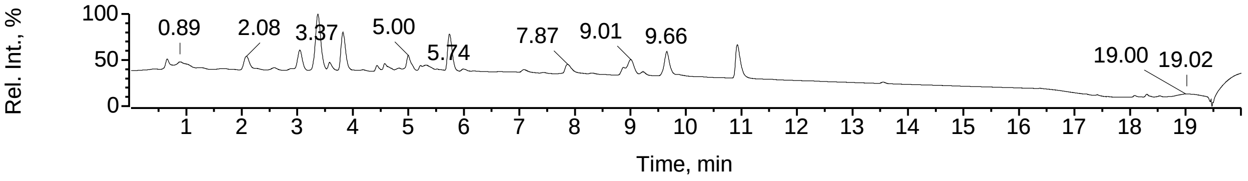

MS SPECTRUM

-MS at 4.20 min

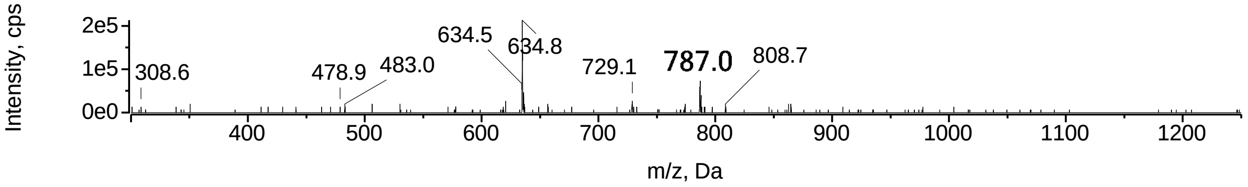

MS/MS COMPARISON WITH PARENT

(Parent Retention Time: 5.38 minutes)

-MSMS of 787.1 (-galloyl)

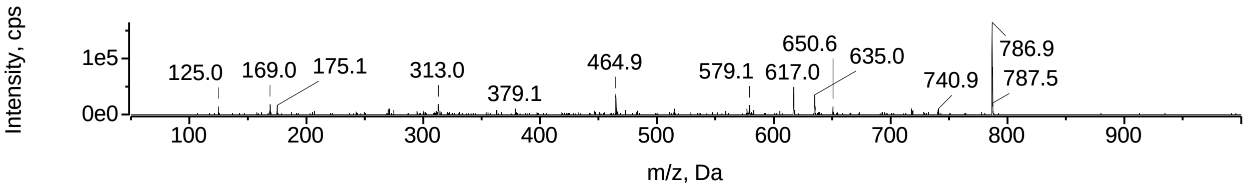

-MSMS of 939.1 (Parent)

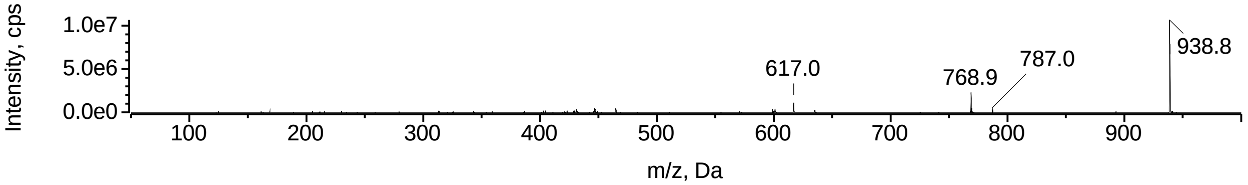

Common product ions (Min. Relative Intensity Shown: 5 %)

168.8, 464.8, 617.2, 634.8, 635.3, 787

Common losses (Min. Relative Intensity Shown: 5 %)

Loss	Metabolite m/z	Parent m/z
-152.2	634.9	787.0
-151.7	635.4	787.3
-0.3	786.8	938.8

M11_Tetragalloyl glucose

-galloyl (-152) in experiment #1: -NL(169.90)
Expected m/z: 787.1
Found m/z: 787.1

	Retention Time	Peak Area
MS	6.24	1.59e6

CHROMATOGRAM (XIC)

XIC of 787.1 (-galloyl/-152.0)

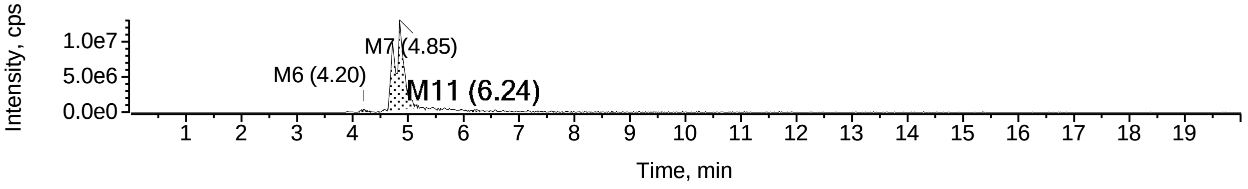

ANALOG CHROMATOGRAM (XWC)

Analog Data

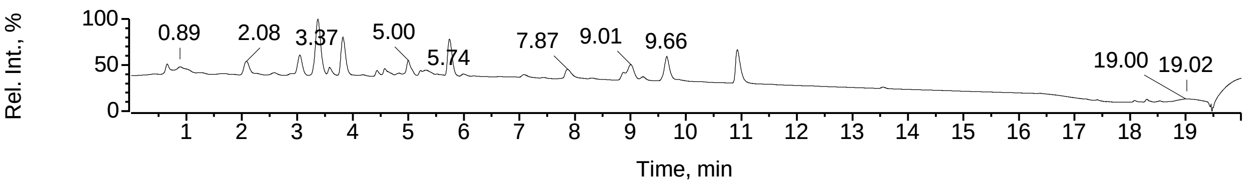

MS SPECTRUM

-MS at 6.24 min

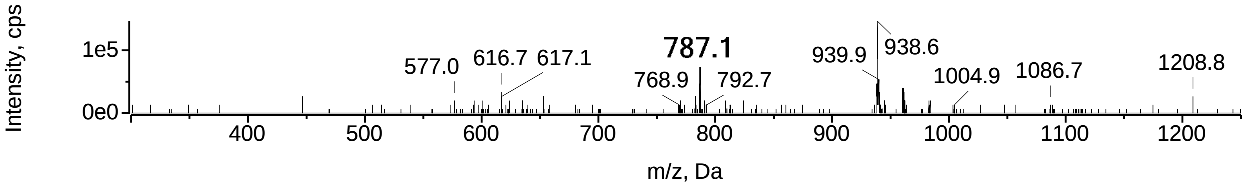

MS/MS COMPARISON WITH PARENT

(Parent Retention Time: 5.38 minutes)

-MSMS of 787.1 (-galloyl)

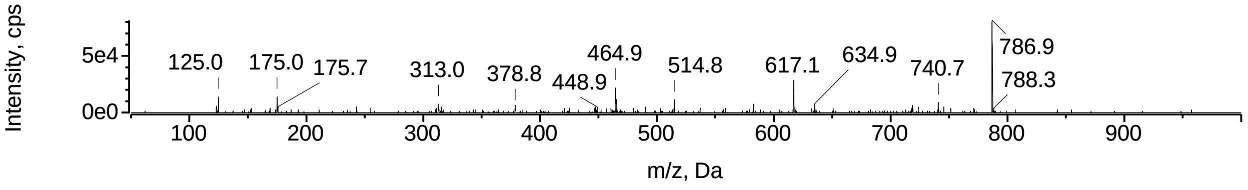

-MSMS of 939.1 (Parent)

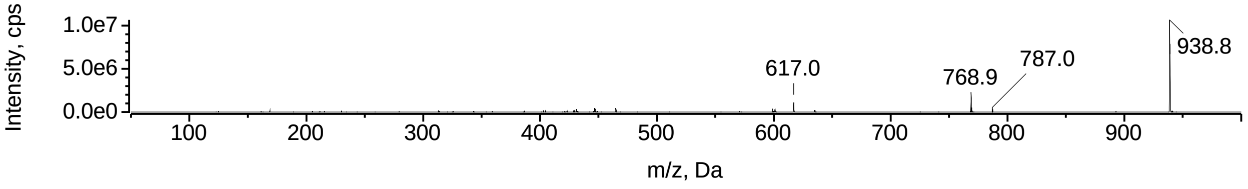

Common product ions (Min. Relative Intensity Shown: 5 %)

464.8, 617.2, 634.8, 787

Common losses (Min. Relative Intensity Shown: 5 %)

Loss	Metabolite m/z	Parent m/z
-152.2	634.9	787.0
-0.3	786.8	938.8

M10_Tetragalloyl glucose

-galloyl (-152) in experiment #1: -NL(169.90)
Expected m/z: 787.1
Found m/z: 786.9

	Retention Time	Peak Area
MS	5.74	3.08e6
Analog	5.74	2.31e2

CHROMATOGRAM (XIC)

XIC of 787.1 (-galloyl/-152.0)

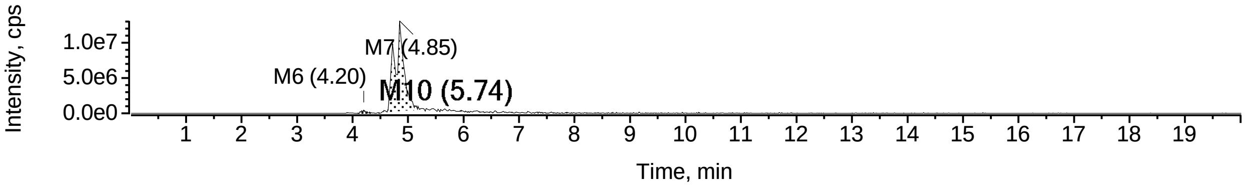

ANALOG CHROMATOGRAM (XWC)

Analog Data

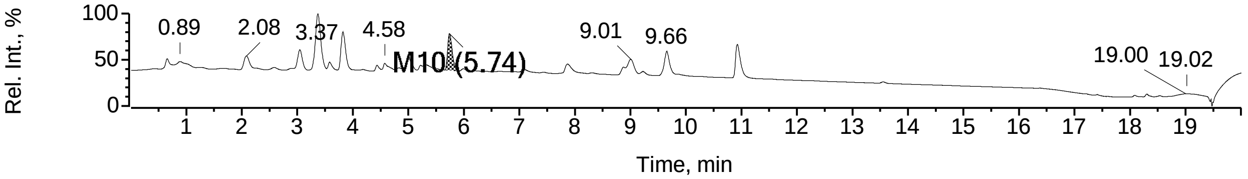

MS SPECTRUM

-MS at 5.74 min

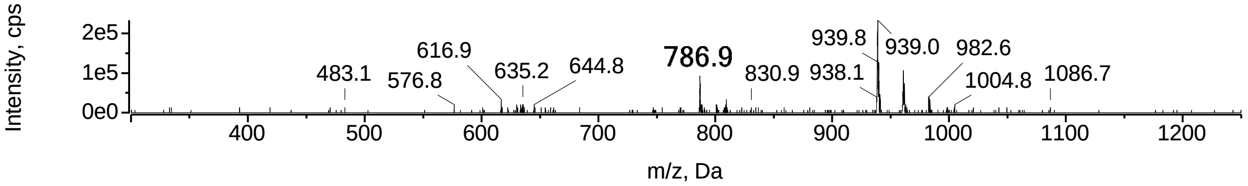

MS/MS COMPARISON WITH PARENT

(Parent Retention Time: 5.38 minutes)

-MSMS of 787.1 (-galloyl)

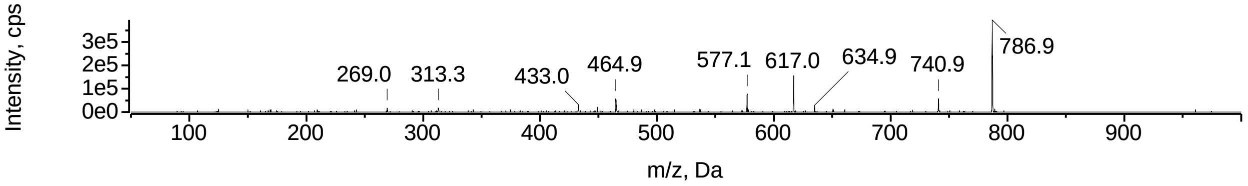

-MSMS of 939.1 (Parent)

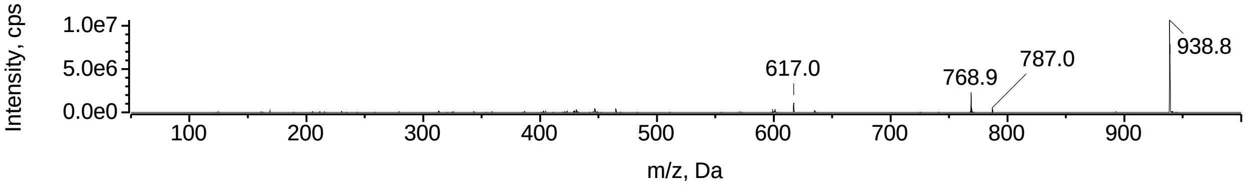

Common product ions (Min. Relative Intensity Shown: 5 %)

464.8, 634.8, 787

Common losses (Min. Relative Intensity Shown: 5 %)

Loss	Metabolite m/z	Parent m/z
-474.3	312.8	464.8
-338.1	449.0	601.1
-170.2	616.9	768.8

M7_Tetragalloyl glucose

-galloyl (-152) in experiment #1: -NL(169.90)
Expected m/z: 787.1
Found m/z: 786.7

	Retention Time	Peak Area
MS	4.85	1.58e8
Analog	4.83	2.23e1

CHROMATOGRAM (XIC)

XIC of 787.1 (-galloyl/-152.0)

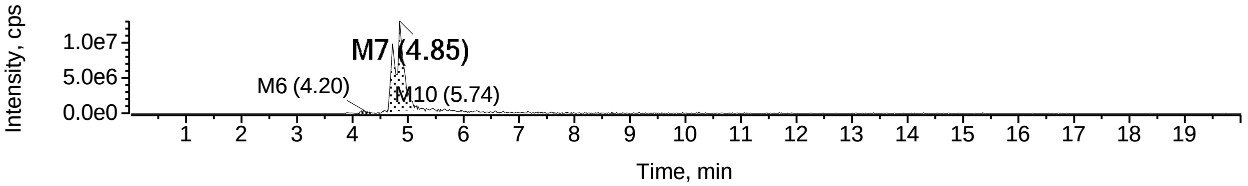

ANALOG CHROMATOGRAM (XWC)

Analog Data

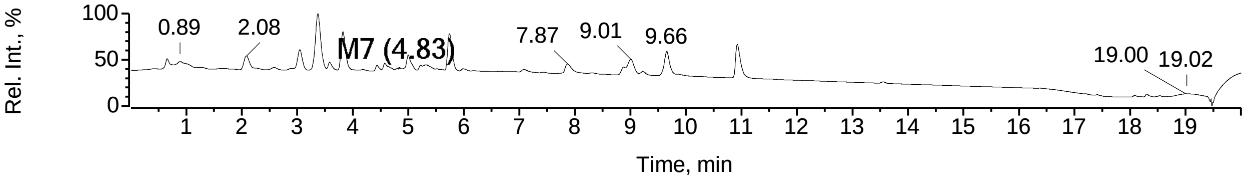

MS SPECTRUM

-MS at 4.85 min

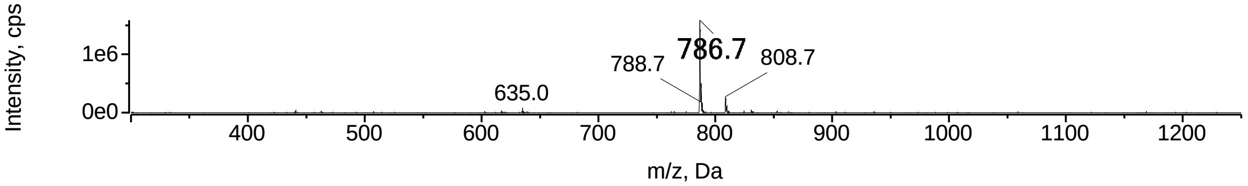

MS/MS COMPARISON WITH PARENT

(Parent Retention Time: 5.38 minutes)

-MSMS of 787.1 (-galloyl)

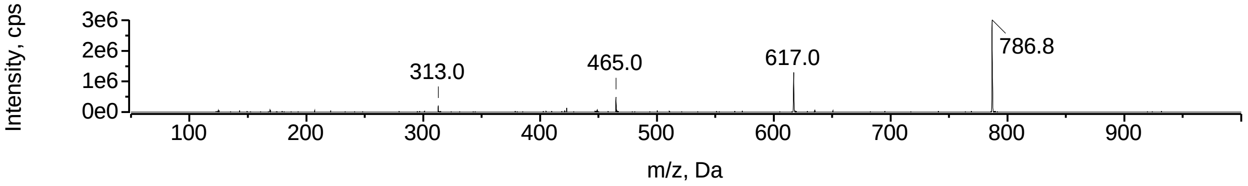

-MSMS of 939.1 (Parent)

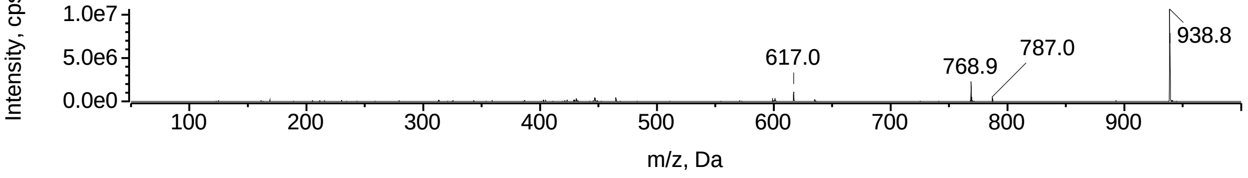

Common product ions (Min. Relative Intensity Shown: 5 %)

617.2, 787

Common losses (Min. Relative Intensity Shown: 5 %)

Loss	Metabolite m/z	Parent m/z
-0.3	786.8	938.8

M2_Digalloyl glucose

-trigalloyl (-456) in experiment #1: -NL(169.90)

Expected m/z: 483.1

Found m/z: 483.2

	Retention Time	Peak Area
MS	2.22	3.33e6
Analog	2.08	1.15e2

CHROMATOGRAM (XIC)

XIC of 483.1 (-trigalloyl/-456.0)

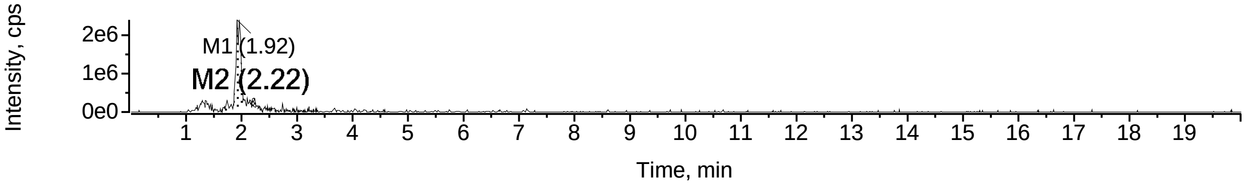

ANALOG CHROMATOGRAM (XWC)

Analog Data

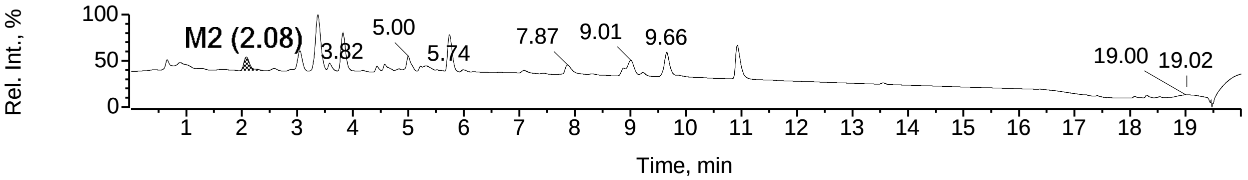

MS SPECTRUM

-MS at 2.22 min

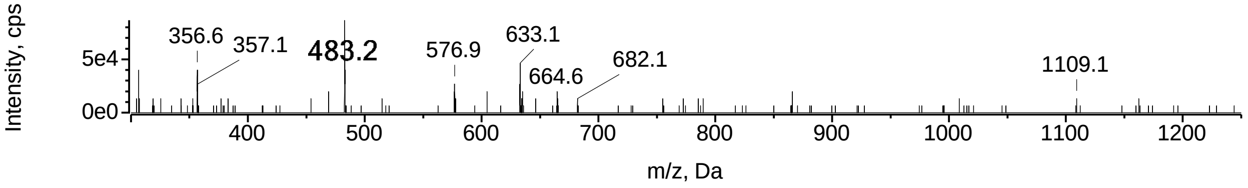

MS/MS COMPARISON WITH PARENT

(Parent Retention Time: 5.38 minutes)

-MSMS of 483.1 (-trigalloyl)

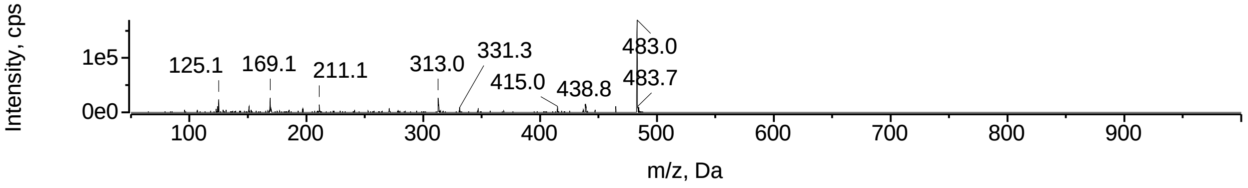

-MSMS of 939.1 (Parent)

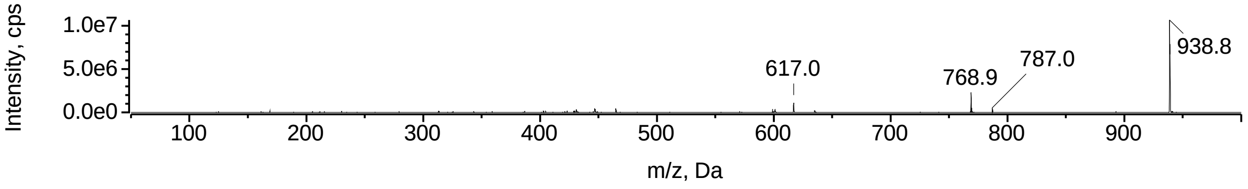

Common product ions (Min. Relative Intensity Shown: 5 %)

447.1, 464.8

Common losses (Min. Relative Intensity Shown: 5 %)

Loss	Metabolite m/z	Parent m/z
-170.3	312.8	768.8
-152.1	331.0	787.0
-151.8	331.3	787.3

M1_Digalloyl glucose

-trigalloyl (-456) in experiment #1: -NL(169.90)

Expected m/z: 483.1

Found m/z: 483.0

	Retention Time	Peak Area
MS	1.92	1.71e7

CHROMATOGRAM (XIC)

XIC of 483.1 (-trigalloyl/-456.0)

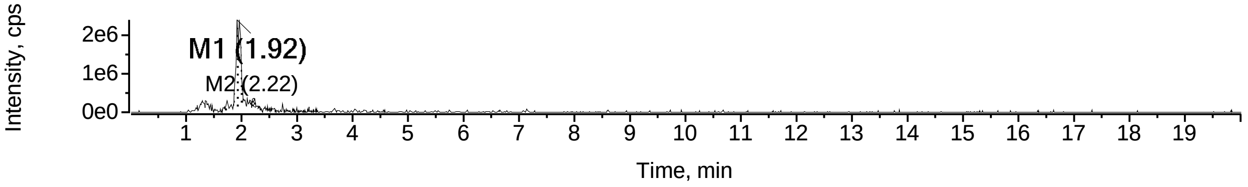

ANALOG CHROMATOGRAM (XWC)

Analog Data

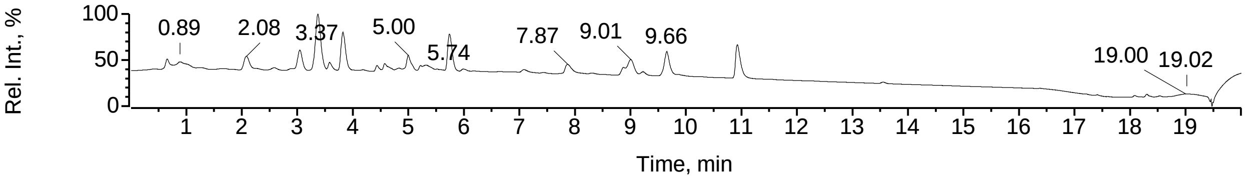

MS SPECTRUM

-MS at 1.92 min

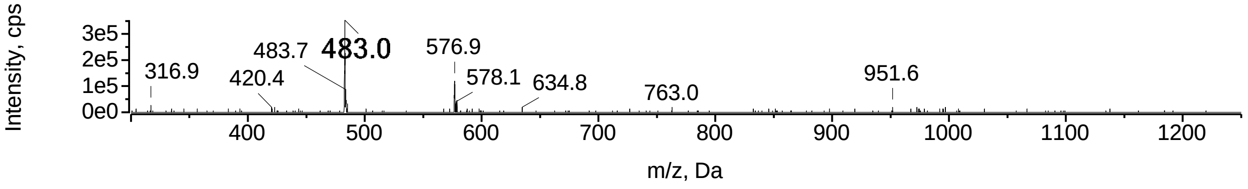

MS/MS COMPARISON WITH PARENT

(Parent Retention Time: 5.38 minutes)

-MSMS of 483.1 (-trigalloyl)

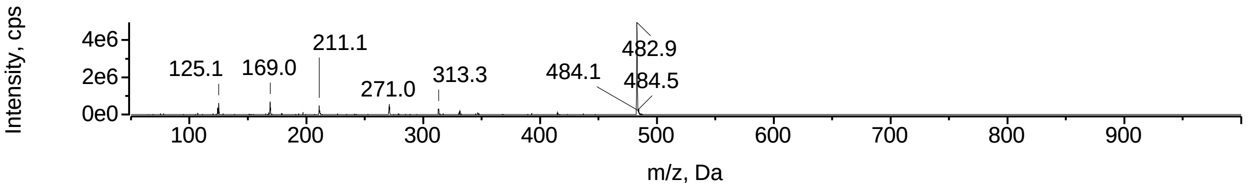

-MSMS of 939.1 (Parent)

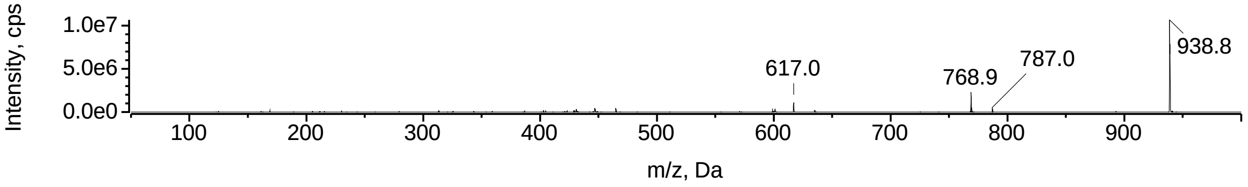

Common product ions (Min. Relative Intensity Shown: 5 %)

313.2

Common losses (Min. Relative Intensity Shown: 5 %)

Loss	Metabolite m/z	Parent m/z
-0.2	482.9	938.8
