# Supplementary material for: A Novel Strategy for Unveiling Spatial Distribution Pattern of Gallotannins in Paeonia rockii and Paeonia ostii Based on LC–QTRAP–MS
Source: Metabolites. 2022 Apr 4;12(4):326. doi: 10.3390/metabo12040326 (PMC9030617; doi:10.3390/metabo12040326)

MS/MS spectrum of glucogallin

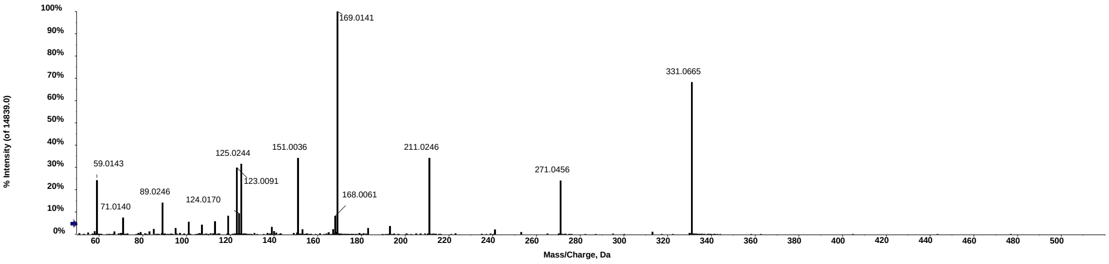

MS/MS spectrum of glucogallin

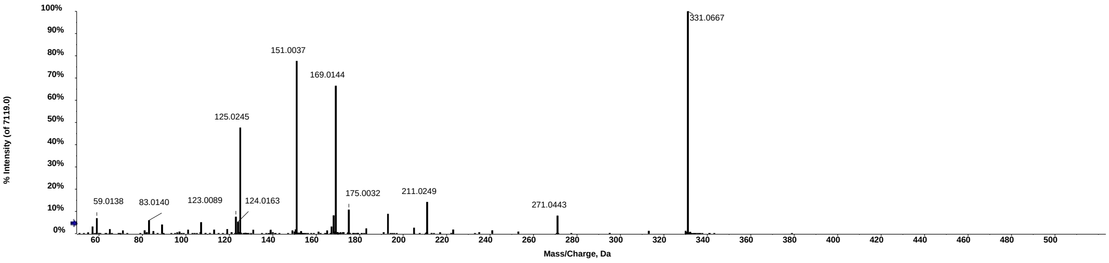

MS/MS spectrum of digalloyl glucose

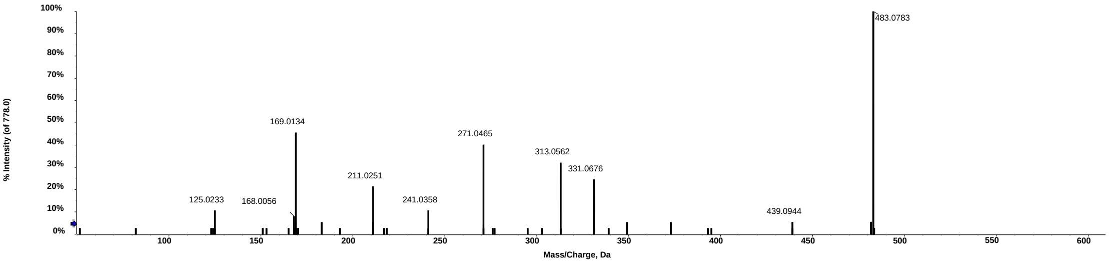

MS/MS spectrum of trigalloyl glucose

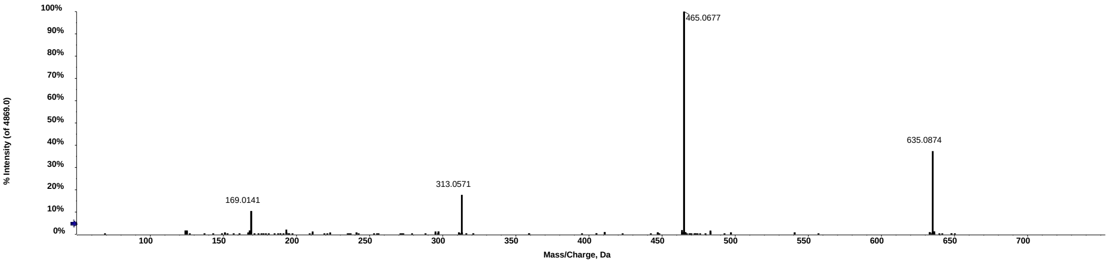

MS/MS spectrum of trigalloyl glucose

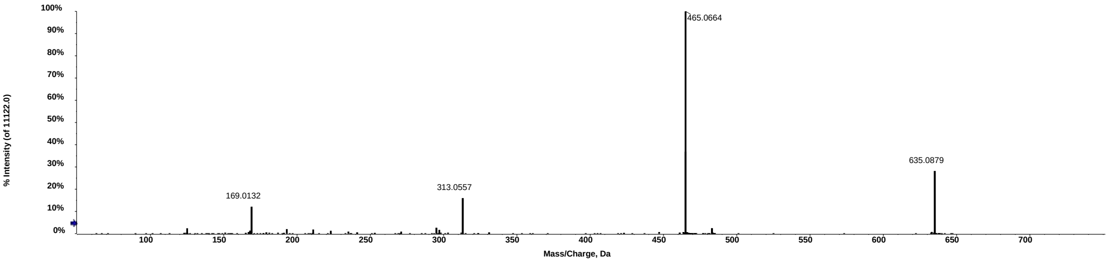

MS/MS spectrum of tetragalloyl glucose

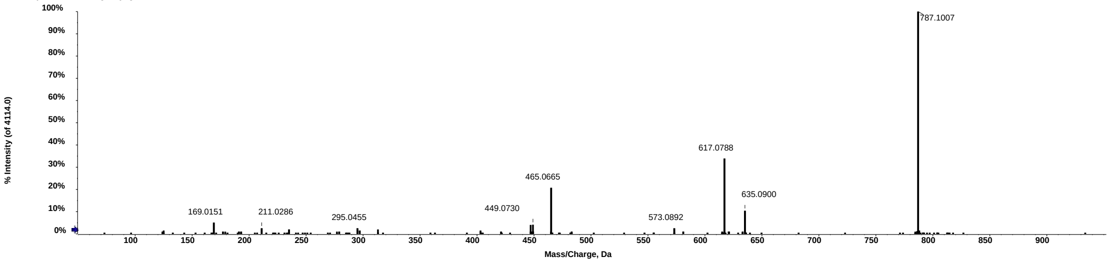

MS/MS spectrum of tetragalloyl glucose

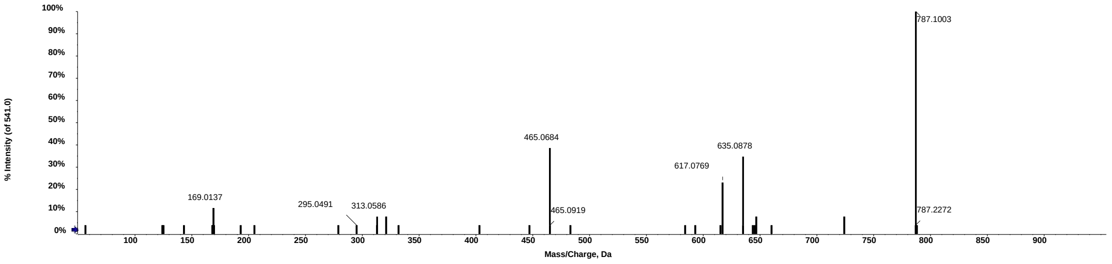

MS/MS spectrum of pentagalloyl glucose

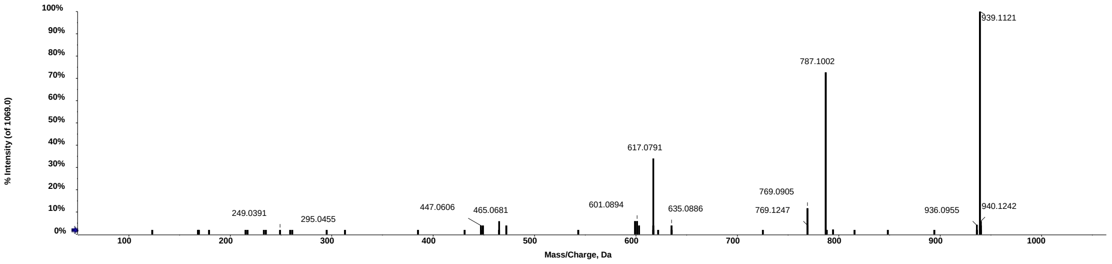

MS/MS spectrum of hexagalloyl glucose

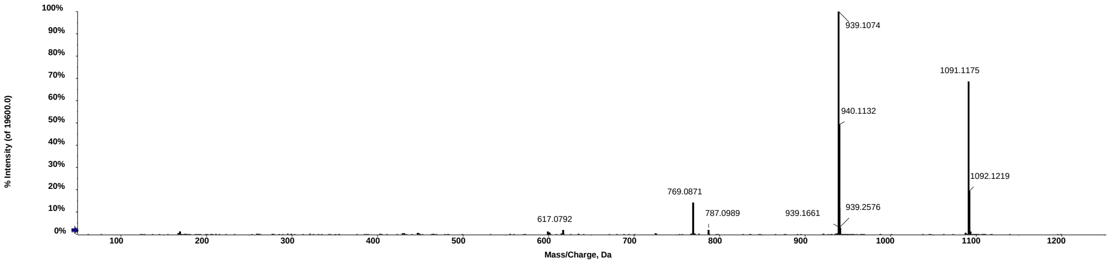

MS/MS spectrum of heptagalloyl glucose

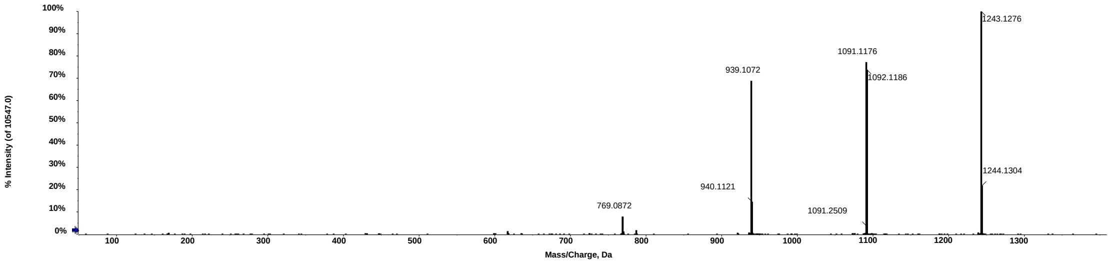

Supplement: Supplementary file 1 [file metabolites-12-00326-s001.zip › Supplementary File S2.pdf]
