# Supplementary material for: A Novel Strategy for Unveiling Spatial Distribution Pattern of Gallotannins in Paeonia rockii and Paeonia ostii Based on LC–QTRAP–MS
Source: Metabolites. 2022 Apr 4;12(4):326. doi: 10.3390/metabo12040326 (PMC9030617; doi:10.3390/metabo12040326)
Supplement: Supplementary file 1 [file metabolites-12-00326-s001.zip › Supplementary materials.pdf]

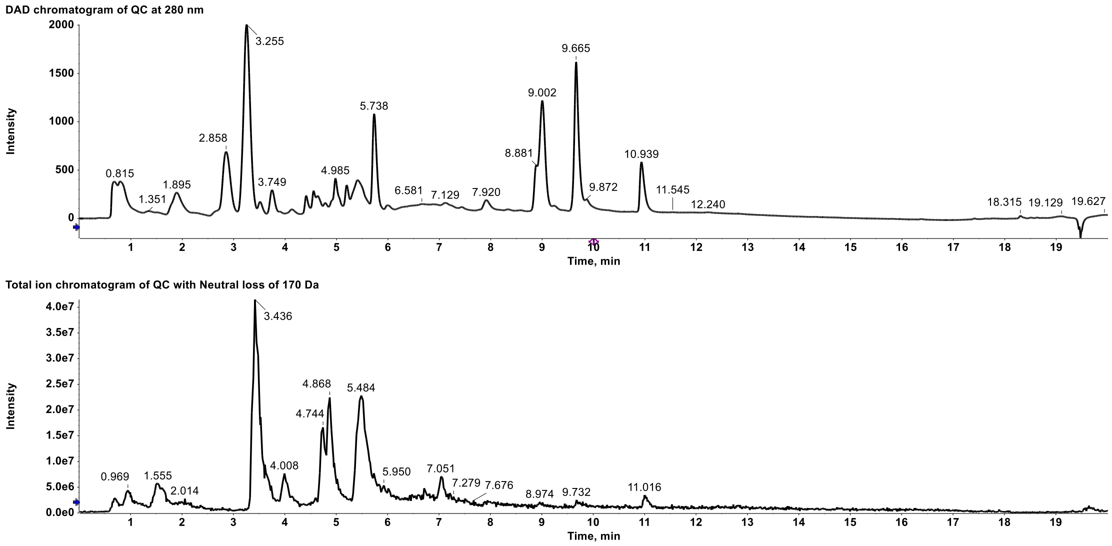

Figure S1. DAD and TIC chromatograms of QC obtained with optimized chromatographic conditions.

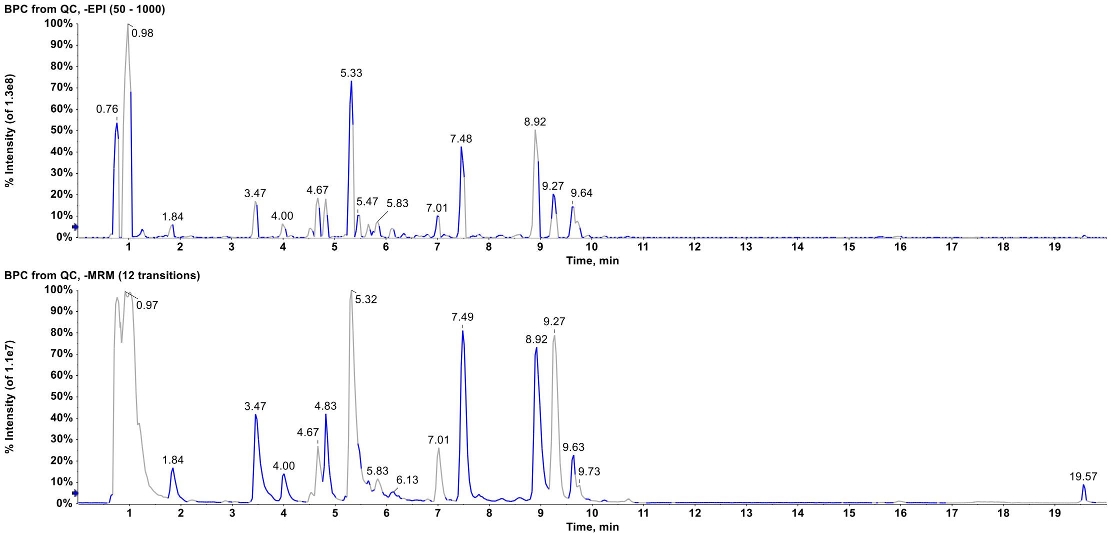

Figure S2. BPC chromatograms of QC sample acquired from MRM-IDA-EPI analysis.

Table S1. Optimized MRM parameters of targeted gallotannins in tree peony.

NO.	RT	compound name	Q1	Q3	DP	CE
1	0.76	Glucogallin	331.1	169	-60	-30
2	0.96	Glucogallin	331.1	169	-70	-30
3	1.92	Digalloyl glucose	483.1	331.1	-60	-30
3	1.92	Digalloyl glucose	483.1	169	-40	-45
4	3.52	Trigalloyl glucose	635.1	465.1	-70	-30
4	3.52	Trigalloyl glucose	635.1	313	-60	-45
5	4.07	Trigalloyl glucose	635.1	465.1	-30	-30
5	4.07	Trigalloyl glucose	635.1	313	-60	-45
6	4.2	Tetragalloyl glucose	787.1	635.1	-80	-30
6	4.2	Tetragalloyl glucose	787.1	617.1	-90	-35
6	4.2	Tetragalloyl glucose	787.1	465.1	-50	-45
7	4.85	Tetragalloyl glucose	787.1	635.1	-60	-35
7	4.85	Tetragalloyl glucose	787.1	617.1	-40	-40
7	4.85	Tetragalloyl glucose	787.1	465.1	-20	-45
8	5.39	Pentagalloyl glucose	939.1	787.1	-10	-45
8	5.39	Pentagalloyl glucose	939.1	769.1	-10	-45

9	5.74	Tetragalloyl glucose	787.1	635.1	-100	-35
9	5.74	Tetragalloyl glucose	787.1	617.1	-20	-40
9	5.74	Tetragalloyl glucose	787.1	465.1	-20	-45

Table S2. The coefficient of variation (CV) of targeted gallotannins.

NO.	RT	compound name	CV of retention time (%)	CV of peak area (%)
1	0.76	Glucogallin	0.98	1.83
2	0.96	Glucogallin	0.73	2.03
3	1.92	Digalloyl glucose	0.82	1.12
4	3.52	Trigalloyl glucose	0.22	0.42
5	4.07	Trigalloyl glucose	0.19	0.75
6	4.2	Tetragalloyl glucose	0.36	1.62
7	4.85	Tetragalloyl glucose	0.46	0.65
8	5.39	Pentagalloyl glucose	0.15	3.60
9	5.74	Tetragalloyl glucose	0.27	5.05
